# Systems biology of resurrection plants

**DOI:** 10.1007/s00018-021-03913-8

**Published:** 2021-08-14

**Authors:** Tsanko Gechev, Rafe Lyall, Veselin Petrov, Dorothea Bartels

**Affiliations:** 1 Center of Plant Systems Biology and Biotechnology, 139 Ruski Blvd., Plovdiv, 4000 Bulgaria; 2 Department of Plant Physiology and Molecular Biology, University of Plovdiv, 24 Tsar Assen Str., Plovdiv, 4000 Bulgaria; 3 Department of Plant Physiology, Biochemistry and Genetics, Agricultural University - Plovdiv, 12, Mendeleev Str, Plovdiv, 4000 Bulgaria; 4 IMBIO University of Bonn, Kirschallee 1, Bonn, 53115 Germany

**Keywords:** Desiccation tolerance, Early light-inducible proteins, Resurrection plants

## Abstract

Plant species that exhibit vegetative desiccation tolerance can survive extreme desiccation for months and resume normal physiological activities upon re-watering. Here we survey the recent knowledge gathered from the sequenced genomes of angiosperm and non-angiosperm desiccation-tolerant plants (resurrection plants) and highlight some distinct genes and gene families that are central to the desiccation response. Furthermore, we review the vast amount of data accumulated from analyses of transcriptomes and metabolomes of resurrection species exposed to desiccation and subsequent rehydration, which allows us to build a systems biology view on the molecular and genetic mechanisms of desiccation tolerance in plants.

## Introduction

Resurrection plants are a unique group of species that can survive dehydration to an air-dried state for months, losing most of their cellular water, and quickly resume normal physiological activities after rehydration [1]. The term “resurrection plants” is most often used to describe vascular desiccation-tolerant species, but this phenotype can be observed across all green plants [1, 2]. A highly efficient protection system has evolved in these species to deal with the dehydration-induced irreversible damage which occurs in desiccation-sensitive plants. Drought can trigger premature senescence in sensitive species which does not normally happen in resurrection plants [3]. The vegetative desiccation tolerance (VDT) strategy used by resurrection species to survive water deficiency is fundamentally different from that employed by succulents and ephemeral plants, which cope with drought by maintaining high water potential and avoidance of drought periods, respectively [2, 4].

**Fig. 1:**
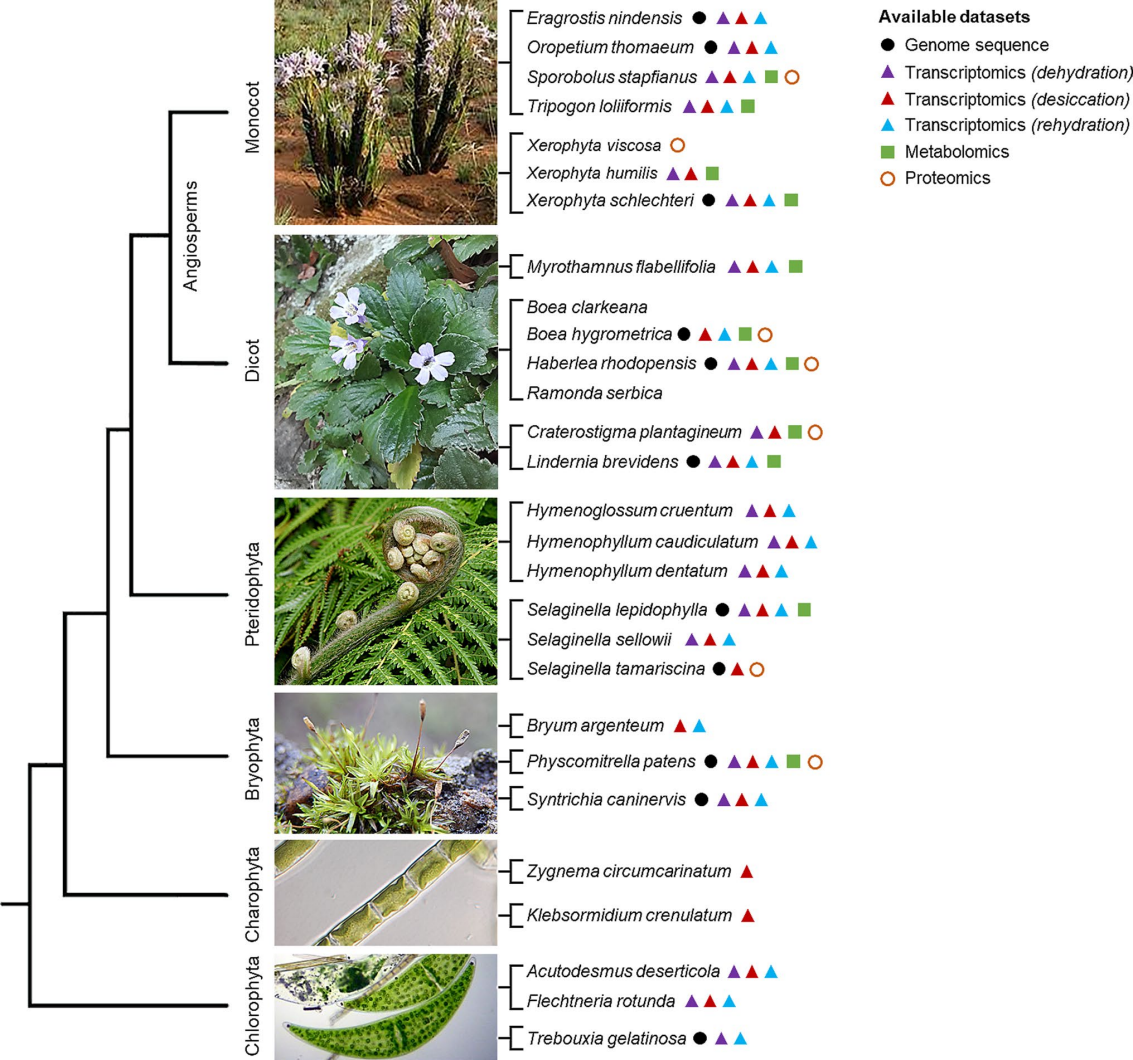
Overview of available next-generation experimental datasets across resurrection species. The relationship between the land plant lineages is displayed as a tree. The symbols beside each species indicate the availability of published data: genome (black circles), gene expression data (coloured triangles) and/or metabolomic data (green squares). Vascular plants (pteridophytes and angiosperms) are the most broadly investigated desiccation-tolerant species, although the trait is more common in non-tracheophytes. Nonetheless, experimental data for these species are currently restricted to a few families (indicated by grouped branch tips): Poaceae and Velloziaceae (monocots), Myrothamnaceae, Gesneriaceae and Linderniaceae (dicots), and Hymenophyllaceae and Selaginellaceae (ferns and lycophytes)

Although resurrection plants are a relatively small group, they exhibit wide taxonomic, ecological, and geographic diversity (Fig. 1). Desiccation-tolerant species comprise algae, mosses, ferns, and lycopods, as well as angiosperm plants (both monocots and dicots), but not in gymnosperms [1, 5]. About 300 angiosperm resurrection plants have been described evolving independently in at least 13 different lineages making up less than 0.2% of the total Flora [6, 7]. Although most resurrection plants are found in habitats with seasonal dry periods, a few species are found in areas where drought is not a common stress. The species *Lindernia brevidens*, for example, grows even in tropical rain forests that lack a dry period [1, 8]. Geographically, resurrection species are found in almost all continents (Africa, Australia, Asia, Europe, the Americas). Africa, especially Southern Africa, is particularly rich in resurrection species [1].

The protection system in desiccation-tolerant plants represents an intricate network of multigenic and multi-factorial processes which modulate a wide array of physiological, morphological, cellular, transcriptomic, proteomic, and metabolic processes designed to counteract dehydration-induced damage and to facilitate the return to homeostasis after rehydration [9, 10]. The term “desiccomics” has been proposed to summarize “-omics” technologies applied to desiccated tissues to unravel this molecular network [11].

Advances in DNA sequencing technologies have facilitated genome studies which have uncovered the genomes of a number of resurrection species and enabled comparative genomics with desiccation-sensitive ones [12–14]. This was substantiated by numerous transcriptome analyses of resurrection plants during desiccation and subsequent rehydration. In addition, targeted and untargeted metabolomics of desiccation-tolerant and relative desiccation-sensitive species exposed to dehydration and rehydration have further substantiated the findings from the genome and transcriptome studies [15, 16]. Metabolome analyses have also identified unique metabolites specific for resurrection plants, some of which with potential applications in medicine and cosmetics [17–19].

A survey of the available data suggests that the “desiccome” consists of a “core” response, for example the high-level expression of late embryogenesis abundant (LEA) genes, as well as additional species-specific responses. Some resurrection species possess other unique physiological features: for example, *Haberlea rhodopensis* is a perennial plant that can also tolerate long-term darkness of up to several months and freezing temperatures during winter [20, 21].

Here we review these recent studies, providing a systems biology approach towards our understanding of desiccation tolerance.

## Genomics of resurrection plants

The number of VDT species with sequenced genomes has grown steadily over the past few years, spanning mosses, liverworts, basal angiosperms, monocots and dicots (see Table 1 and references therein). De novo sequencing of plant genomes is a challenging task due to their above-average size and complexity compared to many other organisms. High levels of heterozygosity, frequency of polyploidy and content of repetitive DNA are often features of plant genomes and make sequence assembly particularly hard when using short-read sequencing methods [22]. This has caused plant sequencing projects to lag behind those of more easily assembled organisms or restricted analyses to plants with smaller and less complex genomes. However, the democratisation of long-read sequencing technologies (e.g. PacBio SMRT sequencing) and improved scaffolding approaches (e.g. Hi-C) have made it easier to analyze genomes of non-model plants [23, 24]. Although the currently sequenced resurrection plants do not have large genome sizes on average (500 Mb; Table 1), several are examples of species with high heterozygosity or polyploidy that would have severely limited assembly if using short-read technologies exclusively [14, 25–27]. Most recent resurrection plant genomes have been assembled using either PacBio or a combination of Illumina/PacBio sequencing, and three have leveraged Hi-C technologies to produce chromosome-level assemblies (*S. caninervis*, *L. brevidens* and *O. thomaeum*; Table 1).

**Table 1:** Resurrection plants with sequenced genomes

	Genome size (Mb)	Assembly size (Mb)	N50	GC (%)	Repeats (%)	Coding genes	Orphan genes	Technology	Assembly	References
***Physcomitrium patens***	473.2	473.2	464.9 Kb	33.3	57.7	35,307	7796	Sanger	Arachne	[43, 44]
*Selaginella moelendorfii*	106	212.6	515 Kb	44.4	37.5	22,285	8066	Sanger	Arachne	[45]
***Selaginella lepidophylla***	109	122.5	163.2 Kb	–	33.8	27,204	–	Illumina/PacBio	Canu, Quiver/Pilon	[26]
***Selaginella tamariscina***	150	300.73	201.16 Kb	37.4	60.2	27,761	3568	PacBio	Falcon	[27]
***Syntrichia caninervis***	300	292.2	20.5 Mb	41.3	53.6	16,545	1526	Illumina/Hi-C	Meraculous, HiRise	[40]
***Marchantia Polymorpha***	280	225.8	256.9 Kb	39.1	22	19,138	5821	Sanger, 454, Illumina	Newbler, Arachne	[46]
*Eragrostis tef*	622	576	1.55 Mb	45.6	26.5	68,255	–	Illumina, PacBio, Hi-C	Canu, Pilon	[47]
***Eragrostis nindensis***	1000	986	520 Kb	46.6	29	116,452	–	Illumina, PacBio	Canu, Pilon	[14]
***Oropetium thomaeum***	245	236	2 Mb	45.3	43	28,836	–	Illumina, PacBio, Hi-C	Canu, Quiver/Pilon, Juicer/3d-DNA	[48, 49]
***Xerophyta schlechteri***	295.5	295.5	1.67 Mb	36.5	18	25,425	1372	Illumina, PacBio	SparseAssembler,Falcon,DBG2OLC/SSPACE-LongRead, Sparc/Pilon, PBJelly	[25]
***Boea hygrometrica***	1690	1540	110 Kb	42.3	75.8	49,374	26,124	454, Illumina	Newbler, SSPACE, SOAPdenovo	[12]
***Haberlea rhodopensis***	1370	1270	2.92 Mb	38.1	68.7	44,306	4768	PacBio,Hi-C	Falcon, Canu/Arrow, SALSA	
*Lindernia subracemosa*	250	246	1.9 Mb	39	31	33,344	–	Illumina, PacBio	Canu, Pilon	[13]
***Lindernia brevidens***	270	263.2	18.7 Mb	39.2	34	27,204	–	Illumina, PacBio, Hi-C	Canu,Pilon, Juicer/3d-DNA	[13]

Sequencing and assembly details for currently sequenced resurrection plant species, as well as some sensitive relatives. The desiccation-tolerant species are highlighted in bold. Most recent assemblies have leveraged PacBio long-read sequencing technologies to overcome issues of polyploidy and heterozygosity, using Illumina short-reads for error correction. The three most recently assembled species (*O. thomaeum, L. brevidens* and *S. caninervis*) include Hi-C scaffolding to produce chromosome-level assemblies. Resurrection plants show a broad range of genomic features, including size, GC%, repetitive regions and coding/orphan genes, which are probably associated with their direct evolutionary lineage rather than VDT specifically. For species that have both a draft assembly and an improved assembly, multiple references are given

So far, despite several high-quality assemblies, any characteristic genomic features related to desiccation tolerance have proven elusive. Resurrection plants show no patterns in terms of their genome size, GC%, repetitive regions or coding gene counts compared to desiccation-sensitive species (Table 1). One reason for this is possibly the still small number of available species, but a more significant factor may be that most plant genomes already have the capacity to encode desiccation tolerance during at least some stages of the life cycle. Nearly all angiosperms produce desiccation-tolerant tissues in the form of orthodox seeds and pollen, for example, which are both associated with complete desiccation [28, 29]. Current transcriptomic evidence suggests that the VDT phenotype in angiosperms is derived from DT found in seeds because many protective mechanisms and pathways are identical in seeds and desiccation-tolerant vegetative tissues [5, 30]. The consensus is that these seed networks ultimately originate from the ancestral VDT mechanisms found in the tissues and spores of early land plants, and which still exist in some form in basal land plants [6, 31]. This suggests that the protective genes and signalling pathways required for the VDT phenotype are already present in the genomes of most plants: the seed maturation networks in angiosperms and, in non-seed plants, the ancestral DT networks from which they were derived. In angiosperms, even the small percentage of species that have recalcitrant (desiccation sensitive) seeds appear to still encode most of the genes required for seed maturation (such as LEAs, oleosins, caleosins and SSPs) and seed maturation itself is simply disrupted or skipped [32, 33]. Manipulation of seed hormonal signalling (application of ABA or suppression of GA) can re-induce DT in some recalcitrant seeds [33, 34]. In the case of *Citrus limon*, reversion to the DT phenotype was not associated with up-regulation of protective mechanisms but rather with suppression of some metabolic pathways, suggesting that these seeds not only can express DT-associated genes but do so under normal maturation conditions [33]. The maintenance of core DT-related genes in the genomes of species with recalcitrant seeds may be because many of the DT protection mechanisms perform a dual function relevant to recalcitrant seed success (for example, LEAs protect against multiple, non-desiccation stresses), or because these mechanisms are nonetheless still required for the development of DT pollen. As such, it seems that the independent re-occurrence of the VDT phenotype across land plants has involved some lineage-specific adjustments to the existing DT mechanisms, rather than evolution of novel pathways. So far, the most detailed analyses of the genomic factors that are common to resurrection plants have focused on late embryogenesis abundant (LEA) proteins, due to their diversity and seemingly intrinsic association to the VDT phenotype across organisms, and early light-inducible proteins (ELIPs) [35, 36]. Resurrection species are also known to encode species- or lineage-specific genes, many of which may be differentially regulated during desiccation [12, 37–40] (Table 1). However, in general, high numbers of orphan genes are not uncommon in plants and it seems unlikely that the complex, multigenic VDT trait could have re-evolved independently numerous times via entirely novel proteins [41, 42].

### LEAs

LEA proteins were first identified as accumulating in the tissues of maturing seeds and are some of the most highly abundant transcripts in desiccating tissues of both seeds and resurrection plants [39, 50–52]. The importance of LEAs or LEA-like proteins in regard to desiccation has been observed not only in plants but in all DT organisms—from bacteria to animals [53–56]. Arabidopsis mutant seeds that show sensitivity to desiccation show dysregulation of some LEAs and an absence of LEAs is associated with the recalcitrant seed phenotype [57–59]. Nonetheless, not all LEAs appear to be linked to DT specifically. Silencing dehydrin genes in Arabidopsis seeds has no impact on DT, and dehydrins are more robustly up-regulated in some recalcitrant seeds than in orthodox relatives.[57, 60]. LEAs have been implicated in responses to a diverse range of abiotic stresses (including drought, heat, cold/freezing and salinity), so it seems likely that specific LEAs or LEA families may be involved with specific stresses other than desiccation [52, 61]. LEA proteins up-regulated during desiccation in resurrection plants probably help defend against not only the direct stresses of water loss but also associated, indirect stresses such as temperature, high light and ROS.

LEAs have been divided into several groups based on the occurrence of conserved domains or other features [62–64] (Table 2). Eight of these groups are associated with defined PFAM domains and have been analysed in detail across land plants, including resurrection species [35]. Analysis of the evolutionary origin of LEA proteins suggests that at least the SMP and LEA_5 family were present in the ancestors of land plants. The other LEA groups may have arisen later in bryophytes (Dehydrin, LEA_2 and LEA_4) and lycophytes (LEA_1 and LEA_3). Only the LEA_6 family is found specifically in angiosperms [35]. The diversity of the LEA family has made it hard to pin down a specific function for these proteins during desiccation and other abiotic stresses. Evidence also suggests that no single function is universal across all LEAs, given the broad range of mutant phenotypes, interactions and activities attributed to specific LEAs from different families or even within the same family [61, 63]. All LEAs share some conserved structural similarities which may shed light on their function(s) during desiccation (for reviews, see [52, 65, 66]). LEAs are highly hydrophillic, with a biased amino acid composition which results in an intrinsically disordered structure. They are predicted to assume a random coil structure in solution but have been shown to sometimes form stable secondary structures under stress. The low number of intra-molecular hydrogen bonds may provide a proportionally large surface area with which to interact with water, other proteins, or cellular structures. LEAs may thus act as “water replacement” proteins, binding to proteins and other molecules that have lost a hydration shell, stabilising their structure, and preventing interaction with other cellular components, preventing molecular crowding. Alternatively (or additionally) LEAs may bind water, promoting the maintenance of existing hydration shells and “buffering” local water loss. LEAs have also been suggested to act as molecular chaperones or to have a role in sequestering metal or non-metal ions. As LEAs may only adopt certain secondary structures under distinct conditions (or water contents), it is also possible that they are only functional during stress—or that their function adapts to increasing or alternate stresses. The wide range of LEA protein expression in plants, coupled with the incredibly broad range of putative functions, makes it hard to specify the exact biochemical role they play during VDT specifically.

**Table 2:** Number of LEA genes from each of the eight LEA families in several resurrection species

	Total	DHN	LEA_1	LEA_2	LEA_3	LEA_4	LEA_5	LEA_6	SMP	Reference
***Physcomitrium patens***	47	3	0	26	0	12	3	0	3	[68]
*Selaginella moelendorfii*	36	2	1	19	0	2	8	0	4	[26]
***Selaginella lepidophylla***	65	4	3	31	0	2	14	0	11	[26]
***Selaginella tamariscina***	40	2	2	14	5	6	8	0	3	[27]
***Syntrichia caninervis***	59^a^	2	0	13	1	22	10	0	2	[40]
***Marchantia polymorpha***	49^b^	2	2	14	0	23	5	0	3	
***Eragrostis nindensis***	347^c^	29	14	196	37	25	9	9	28	[14]
*Eragrostis tef*	206^c^	10	12	135	14	13	6	3	13	[14]
***Oropetium thomaeum***	102	8	7	61	7	7	2	1	9	[14]
***Xerophyta schlechteri***	111	14	6	57	4	8	6	6	10	[68]
*Arabidopsis thaliana*	83	9	3	46	4	10	2	3	6	[68]
***Boea hygrometrica***	55	5	6	33	2	2	1	2	4	[27]
***Haberlea rhodopensis***	115^b^	10	13	67	4	9	6	1	5	
*Lindernia subracemosa*	83	10	5	42	6	12	2	2	4	[13]
***Lindernia brevidens***	77	9	4	36	5	14	3	2	4	[13]

The number of LEA genes identified in the genomes of several resurrection plants and closely related sensitive species. The desiccation-tolerant species are in bold. LEA family domains are based on classification by PFAM. The reference to the original studies citing the numbers given in this table has been included, though the number of genes per species is not consistent across the current literature

^a^Nine putative LEAs could not be assigned to any of the eight families

^b^Putative LEAs were identified in these species by querying the known LEA PFAM domains against the proteome using *hmm search*

^c^A recent review [1] places the copy number of these genes far lower than the original publication, suggesting subsequent refinement of the genomes or annotations

While most resurrection plants encode large numbers of LEA genes that are preferentially highly expressed in dehydrated tissues, there does not appear to be a conserved pattern of LEA gene family expansion compared to sensitive species [35] (Table 2). Unlike sensitive species, many resurrection plants show constitutively high expression of LEA transcripts in unstressed tissues as well as induction of “seed-specific” LEAs in adult tissues. Conversely, in aquatic angiosperm species that do not produce orthodox seeds or pollen and do not regularly encounter desiccation stress, the LEA gene family is reduced compared to terrestrial species [28, 67]. The lack of consistent LEA family expansion in resurrection plants could simply be because the diverse pool of LEAs that already existed in their sensitive ancestors was sufficient to support the VDT phenotype, requiring only adjustments to LEA gene regulation.

### ELIPs

Currently, only one genomic “signature” for desiccation tolerance has been identified: a conserved, massive expansion of the early light-induced protein (ELIP) gene family. Resurrection plants encode an average of 20.7 ELIP genes, generally spanning large tandem arrays, compared to only 3.1 in sensitive species [36]. ELIPs are part of the light-harvesting complex (LHC) protein superfamily and are localised to the thylakoid membranes. This superfamily consists of classical LHC proteins—the components of the photosynthetic antenna complexes in chloroplasts associated directly with light harvesting—as well as related stress-induced proteins proposed to be involved in photoprotection. These LHC-like proteins include ELIPs, one-helix proteins (OHPs), stress-enhanced proteins (SEPs) and PSBS proteins [69, 70]. ELIPs were first identified during de-etiolation of pea and barley seedlings where they were hypothesised to be involved in chloroplast biogenesis or photoprotection based on their expression profile. Subsequently, they were also identified in mature tissues exposed to high light and other abiotic stresses giving support to a putative photoprotective role [69–71]. These stresses include cyclic heat shock, cold stress, osmotic stress, salt stress, desiccation, low O_2_/CO_2_, oxidative stress, nutrient starvation and UV-B (see [69, 72]. Besides germination, ELIPs are also induced during other development stages that involve chloroplast disruption, such as the chloroplast-chromoplast transition in floral development and ripening fruit, or during leaf senescence [69, 73, 74]. Induction of ELIPs has been previously observed in desiccating resurrection plant tissues across multiple species [36].

There have been several hypotheses regarding ELIP function. Because the structure of ELIPs is similar to that of LHC antennae proteins, one mode of action is thought to be via energy dissipation during photoinhibition [71]. However, there has been no substantial evidence to support this role so far [75–77]. Alternatively, ELIPs may act as transient pigment carriers able to bind free chlorophyll, thus directly preventing the generation of toxic ROS, or for the purpose of stabilising photosystem assembly [75–77]. ELIPs may also be relevant in regulating the synthesis or stability of zeaxanthin during stress, as they have been observed to bind xanthophyll pigments [76, 78, 79]. Lastly, there is some evidence that ELIPs may not function in photoprotection directly but are important regulators of chlorophyll biosynthesis by sensing disorganised chlorophyll levels [77, 78, 80]. However, the precise function of the ELIP proteins—and the degree of redundancy with other LHC-like proteins, such as OHPs and SEPs—is still unclear. Whereas OHPs and SEPs are constitutively expressed during low-light conditions and upregulated during high light, ELIPs are specifically transiently upregulated during high light and other stresses but otherwise remain at undetectable levels [69, 70, 81].

The lack of clarity on ELIP functions makes it difficult to speculate on the exact role of ELIPs during desiccation. Interestingly, the duplication of only ELIPs in resurrection species—and not OHPs or SEPs—implies that their function during desiccation tolerance is both specific and non-redundant with the other stress-induced LHC-like proteins [47]. Although ELIPs are classically associated with light stress, upregulation of some ELIPs can be independent of light. In both barley and the green alga *C. reinhardtii*, ELIPs are upregulated in the cold in the absence of light stress [82, 83]. Neither of the two desiccation-induced *ELIP* genes in the bryophyte *S. ruralis* were responsive to high irradiance alone [72]. The Balkan resurrection plant *H. rhodopensis* is extremely tolerant to extended darkness and shows high upregulation of multiple ELIPs even after long-term growth and desiccation in the absence of light [21]. This may be a lineage-specific trait, as the ELIP protein DSP22 from *C. plantageneum* is not induced during desiccation in the dark [72]. Although the two Arabidopsis ELIP genes are assumed to be functionally redundant, there is evidence that *ELIP1* and *ELIP2* are regulated via different signals, and *elip1* and *elip2* single mutants display opposite phenotypes regarding germination rate under abiotic stresses [80, 84]. In developing seeds, *ELIP2* transcript abundance is the highest at the mature green stage, whereas *ELIP1* is expressed in post-mature green, dry and imbibed seeds [84, 85]. The *ELIP1* gene, but not the *ELIP2* gene, contains a G-box element in the promoter commonly associated with ABA-regulated seed maturation genes [84, 86]. In *X. humilis*, the only resurrection species with available seed expression data, some putative ELIP transcripts are highly up-regulated in the dry seed as well as in desiccated leaves [39]. ELIPs also appear to be under strict translational or post-translational control [81]. Only the ELIP1 protein is detected in Arabidopsis leaves exposed to photooxidative stress at 22 °C, despite the induction of both ELIP transcripts [80]. In barley etioplasts, detection of ELIP proteins in the thylakoid membranes occurs after transcript abundance has decreased considerably [87]. The possible disconcordance between transcript and protein abundance in desiccating tissues has been highlighted previously in relation to resurrection species [1, 5, 88].

There appear to be differences in ELIP induction in homoiochlorophyllous and poikilochlorophyllous resurrection species [47]. In homoiochlorophyllous species, ELIP transcript abundance is generally high in desiccated tissues and during early rehydration. In contrast, in poikilochlorophyllous species, ELIP transcript abundance in dry tissues is generally lower than in the preceding stages of desiccation and increases again during rehydration. Interpreting these results is heavily dependent on understanding how ELIP protein levels change during desiccation and rehydration and is further hampered by the small number of sequenced plants and limited time-course expression data for the available species. Some aspects of chloroplast regeneration during rehydration in some resurrection plants is independent of transcription, suggesting that the proteins are already available or are de novo translated from existing transcripts [89]. At least in the case of the homoiochlorophyllous species, *C. plantageneum*, the DSP22 ELIP protein has been shown to be associated with thylakoid membranes in tissue desiccated for 24 h [79]. High ELIP transcript levels could thus be associated with high ELIP protein levels—and thus ELIP protein function—throughout desiccation until rehydration in homoiochlorophyllous species. Poikilochlorophyllous species degrade chlorophyll during desiccation, potentially minimising the effects of photooxidative damage and thus the requirements for ELIP proteins—as evidenced by the lower average number of ELIP genes in these species [47]. Both *Xerophyta* species show a sharp increase in ELIP transcript abundance between 40 and 20% relative water content (RWC), roughly coinciding with the onset of photoinhibition [39, 47, 90]. The subsequent reduction in ELIP transcript levels in dried tissues could suggest that ELIP function during desiccation is limited to this small window, and that ELIP mRNAs are not stored. Stable ELIP insertion into the thylakoids is post-translationally regulated by chlorophyll *a* in barley—increasing linearly with chlorophyll concentration [81, 87]. The highest ELIP expression in both *Xerophyta* species occurs at the point at which chlorophyll is almost fully degraded, though whether this has any influence on ELIP function in these species is unknown [90]. Since the rehydration and regreening of dried poikilochlorophyllous tissues mirrors seedling de-etiolation, re-induction of ELIPs during this period in such resurrection plants may simply be analogous to the process seen in germinating seedlings.

Far more research is required to unravel the function of ELIP proteins during desiccation in resurrection plants and, indeed, in sensitive species. The role they play must be central to the VDT phenotype given the independent expansion of this gene family across all resurrection species analysed so far. The genomic mechanisms required for desiccation tolerance are common to most land plants through the pathways involved in spore, pollen, and seed maturation, and are seemingly sufficient to give rise to the VDT phenotype in sensitive ancestors of resurrection plants given the lack of any other genomic signatures. ELIP function during VDT may be in some way novel compared to their roles during the development and stress response of sensitive species, given that the standard repertoire of ELIP genes appears insufficient across all resurrection species. This could imply direct neofunctionalisation of duplicated ELIPs or simply that the stresses of VDT (particularly related to phototolerance) dwarf those of developmentally regulated desiccation processes found in seeds and similar structures, requiring a larger complement of ELIPs (Table 3).

**Table 3:** Number of ELIP genes in resurrection species

	ELIPs	References
***Physcomitrium patens***	22	[36]
*Selaginella moelendorfii*	2	[36]
***Selaginella lepidophylla***	24	[36]
***Selaginella tamariscina***	74	[36]
***Syntrichia caninervis***	32	[40]
***Marchantia Polymorpha***	28	[36]
***Eragrostis nindensis***	27	[14]
*Eragrostis tef*	5	[14]
***Oropetium thomaeum***	22	[14]
***Xerophyta schlechteri***	9	[36]
*Arabidopsis thaliana*	2	
***Boea hygrometrica***	17	[36]
***Haberlea rhodopensis***	23	Unpublished
*Lindernia subracemosa*	4	[36]
***Lindernia brevidens***	26	[36]

The number of ELIP genes identified in the genomes of several resurrection plants and closely related sensitive species. The desiccation-tolerant species are in bold. The reference gives the publication from which the given value was determined, though exact numbers are different across the literature

## Transcriptomes of resurrection plants

The increased accessibility of next-generation RNA-seq has resulted in increased numbers of resurrection plant transcriptome studies [5]. Most datasets are devoted to dehydration, desiccation and rehydration responses in leaf tissues, but a limited number of experiments also target additional stresses like extended darkness [21], heat stress [91], the influence of hormones like ABA (abscisic acid) and auxins [92, 93], or other organs and developmental stages, including maturing seeds [39] and roots [94]. Transcriptomes of all major taxa in which VDT has been reported have been analyzed (Table 4). The data suggests that there are some core factors related to the DT response, in addition to species- or clade-specific responses which can be attributed to the independent evolutionary origin of the VDT trait [95].

**Table 4:** A summary of transcriptomes of desiccation tolerant species, respective studies and the experimental setup, which are discussed in this chapter

VDT species	References	Conditions
Dicots		
*Boea clarkeana*	[102]	Material collected from the wild
*Boea hygrometrica*	[103]	*C*^*h*^, rapid air desiccation for 2 h (80% RWC) and 48 h (< 10% RWC), gradual desiccation for 5 days (80% RWC) and 14 days (< 10% RWC), rehydration after acclimation (3 days), air-drying after acclimation for 2 and 48 h
	[12]	*C*^*h*^, dehydration (70% RWC), desiccation (10% RWC)
*Craterostigma plantagineum*	[37]	*C*^*h*^, dehydration (48 h, 80% RWC), desiccation (5% RWC), rehydration (24 h)
*Haberlea rhodopensis*	[38]	C^h^, Dehydration (4 days, 42% RWC), desiccation (20 days, 4% RWC), rehydration (4 days)
	[104]	*C*^*h*^, slight dehydration (75% RWC), severe dehydration (20% RWC), desiccation (6% RWC), rehydration (back to 90% RWC)
	[21]	*C*^*u*^, 7 days darkness, 30 days darkness, 7 days recovery in the light
*Myrothamnus flabellifolia*	[105]	*C*^*h*^, slight dehydration (90% initial fresh weight, IFW), severe dehydration (75% IFW), desiccation (27% IFW), earlier rehydration (6 h), later rehydration (12 h)
*Lindernia brevidens*	[13]	*C*^*h*^, mild dehydration (3 days, 53–56% RWC), severe dehydration (7 days, 23–27% RWC), desiccation (10 days and 14 days, 6–9% RWC), earlier rehydration (24 h, ~ 30% RWC), later rehydration (48 h, 44% RWC)
*Ramonda serbica*	[106]	Fully hydrated leaves
Monocots		
*Eragrostis nindensis*	[14]	*C*^*h*^, moderate dehydration (56 h, 68% RWC), severe dehydration (104 h, 15% RWC), desiccation (224 h, 14% RWC), recovery after—0 h, 12 h (81% RWC), 24 h (93% RWC) and 48 h rehydration (86% RWC)
*Oropetium thomaeum*	[107]	*C*^*h*^, dehydration—7 days (31% RWC), 14 days (12% RWC), 21 days (7% RWC) and 30 days (5% RWC); rehydration—24 h (38% RWC) and 48 h (52% RWC)
*Sporobolus stapfianus*	[108]	*C*^*h*^, dehydration—80% RWC, 60% RWC, 40% RWC and 30% RWC, desiccation— ~ 11% RWC, recovery—12 and 48 h after rehydration
*Tripogon loliiformis*	[109]	*C*^*h*^, moderate dehydration (60% RWC), severe dehydration (40% RWC), desiccation (< 10% RWC), rehydration (48 h)
	[94]	*C*^*h*^, moderate dehydration (60% RWC), severe dehydration (40% RWC), desiccation (< 10% RWC), rehydration (48 h) in shoots and roots
*Xerophyta humilis*	[39]	Leaves: *C*^*h*^, dehydration—80% RWC, 60% RWC, 40% RWC, desiccation—5% RWC; seeds—early maturation (DAF6), mature green (DAF11), mature dry (DAF17)
*Xerophyta schlechteri*	[25]	Adult plants: dehydration—*C*^*h*^, 80%, 60%, 40%, 20% and 4% RWC, recovery—12 and 24 h post rehydration; seedlings after a drying/rehydration cycle—shoots and roots treated or not with ABA
Ferns		
*Hymenoglossum cruentum*	[110]	*C*^*h*^, early dehydration (80% RWC), late dehydration (6% RWC), rehydration (80% RWC)
*Hymenophyllum caudiculatum*	[111]	*C*^*h*^, dehydration (~ 60% RWC, 1 day), desiccation (7 days), partial rehydration (~ 60% RWC), full rehydration (~ 90% RWC)
*Hymenophyllum dentatum*	[111]	*C*^*h*^, dehydration (~ 60% RWC, 1 day), desiccation (7 days), partial rehydration (~ 60% RWC), full rehydration (~ 90% RWC)
Lycophytes		
*Selaginella lepidophylla*	[26]	Desiccation, partial recovery (1, 6, and 24 h), full recovery (120 h); second dehydration treatment (24 h)
*Selaginella sellowii*	[112]	*C*^*h*^, dehydration (70% and 50% RWC), desiccation (10% RWC), rehydration (2 and 6 h)
*Selaginella tamariscina*	[113]	*C*^*h*^, moderate dehydration (50% RWC)
Bryophytes		
*Bryum argenteum*	[114]	Dry, rehydration—2 and 24 h
	[115]	*C*^*h*^, dehydration—2 h (~ 25% RWC) and 24 h (< 2% RWC), rehydration—2 and 48 h
*Physcomitrium patens*	[92]	*C*^*u*^, WT and *anrKO* chloronemata treated with ABA, osmotic stress or dehydration (70% RWC)
	[116]	34 different developmental stages and conditions, including: dehydration (30 h after reaching − 13 MPa water potential), and rehydration (2 h)
*Syntrichia caninervis*	[99]	Rehydration of dried gametophores (24 h), de novo dehydration (0.5, 1, 1.5, 2, 4, 6, 8, 10, 12 and 24 h)
	[40]	*C*^*h*^ (at 20 °C), slow drying, rapid drying, rehydration after slow drying (30 min), cold (4 °C for 90 min), elevated temperature (30 °C for 90 min), heat shock (35 °C for 90 min)
Algae		
*Acutodesmus deserticola*	[100]	*C*^*h*^, early dehydration (2.5 h), late dehydration (7.5 h), desiccation (24 h), recovery (1 h after rehydration)
*Flechtneria rotunda*	[100]	*C*^*h*^, early dehydration (2.5 h), late dehydration (7.5 h), desiccation (24 h), recovery (1 h after rehydration)
*Klebsormidium crenulatum*	[117]	*C*^*h*^, desiccation (2.5 h, 7% RWC)
*Trebouxia gelatinosa*	[118]	*C*^*h*^, dehydration (10 h), rehydration (12 h)
*Zygnema circumcarinatum*	[119]	Hydrated and desiccated until Y(II) = 0: liquid culture (1 month), agar plate cultures (7 months)

*C*^*h*^ fully hydrated control, *C*^*u*^ untreated control, *RWC* relative water content, *d* days, *h* hours

Informally, VDT in plants can be one of two forms: constitutive or inducible. Constitutive VDT relies on cellular repair mechanisms triggered after rehydration, which allows survival even after an extremely rapid water loss. In contrast, plants with inducible VDT modify their physiological and biochemical processes during dehydration and thus need sufficient time to prepare an effective protection response [31]. This is not a hard rule—many plants that display inducible VDT nonetheless constitutively express protective mechanisms not found in sensitive plants, and species with constitutive VDT may still require some form of priming or prehardening. Generally, constitutive VDT is common in non-vascular plants (algae and bryophytes) and inducible VDT is found in vascular plants (lycophytes, ferns and angiosperms). This differentiation likely reflects the impact of early colonization of terrestrial ecosystems on the evolution of land plants. The water content of early land plants—and extant non-vascular resurrection plants—is directly linked to the atmospheric water potential, which requires a constitutive protection response to frequent and rapid drying events. The evolution of the vascular system allowed ancestral tracheophytes to regulate water loss, and thus metabolically-costly, constitutive VDT was replaced by inducible mechanisms. VDT was subsequently lost entirely in the ancestors of the angiosperm and gymnosperm lineages, with the ancestral DT mechanisms relegated to the reproductive propagules (seeds and pollen). Consequently, angiosperm resurrection plants, which have re-evolved the VDT trait, are rare—an estimated 330 species or 0.01% of the 380,000 species of tracheophytes described [1, 96]. In comparison, VDT is much more widespread in non-tracheophytes: 210 out of 21,000 known bryophyte species manifest DT features (1%), including 158 mosses, 51 liverworts and one hornwort [97–99]. It is likely that this value is a significant underestimate [1]. While far less studied, VDT is also found in terrestrial green algae, in contrast to their aquatic relatives [100, 101].

Most transcriptomic studies have targeted angiosperm resurrection plants, which are more closely related to agriculturally and commercially valuable crop species than primitive tracheophytes or non-vascular plants (Table 4). The following sections will detail what has been uncovered about the VDT trait from RNA-Seq studies across all these lineages. For brevity, the focus will be on results from next-generation sequencing platforms and not earlier studies.

### Vascular plants

#### Dicots

##### First insights from Craterostigma plantagineum and Haberlea rhodopensis

*Craterostigma plantagineum* was the first VDT plant whose transcriptome was analyzed by NGS [37]. This species was selected as a suitable model system to investigate desiccation since it can be transformed and manifests the VDT trait in both undifferentiated and differentiated tissues. Four distinct stages of the dehydration/rehydration cycle were analyzed in *C. plantagineum*: fully hydrated controls, 48 h dehydration, desiccation and 24 h post rehydration. A similar experimental design was used for the endemic species of the Balkans—*Haberlea rhodopensis*, with the slight difference that the duration of the dehydration treatment was prolonged to 4 days until the relative water content of the leaves dropped to 42% [120]. The recovery was also extended to 4 days.

These time-course evaluations of the transcription profiles in two dicot VDT plants revealed both common and species-specific molecular factors involved in the VDT response (as discussed in Refs. [2] and [121]). For example, some of the most abundant transcripts in the unstressed *C. plantagineum* and *H. rhodopensis* controls were related to photosynthesis as well as cell wall organization and plasticity. Water deprivation led to downregulation of photosynthesis-related transcripts, but upregulation of transcripts encoding late embryogenesis abundant (LEA) proteins, lipocalins, sucrose synthase, pathogenesis-related proteins and some regulatory molecules like NAC transcription factors and phosphatases. Lipocalins are proteins with low molecular mass and simple tertiary structure that can bind small, usually hydrophobic ligands like lipids, steroids, secondary metabolites and others. The two described subgroups of lipocalins in plants, temperature-induced (TILs) and chloroplastic (LCNPs), have been implicated in tolerance to multiple stresses [122]. Although their mechanism of action is not clarified, it has been proposed that lipocalins are able to scavenge hydroperoxy fatty acids and thus prevent lipid peroxidation caused by reactive oxygen species, for example singlet oxygen produced in excess light [123, 124]. Recently Malnoë et al. suggested that LCNPs detoxify peroxidized lipids directly and are required for effective non-photochemical quenching (NPQ), a defense strategy which dissipates excess light [125]. Pathogenesis-related (PR) proteins are a structurally diverse group known to be induced by phytopathogens and exert key functions in the plant innate immune system, especially systemic acquired resistance (SAR). However, despite being traditionally associated with biotic stress, drought and multiple other abiotic cues also activate PR genes [126]. The large NAM/ATAF1/CUC2 (NAC) family of transcription factors (TFs) contain plant-specific proteins that are important in development, senescence, abiotic and biotic stress responses. Therefore, the differential expression of NACs during dehydration/desiccation in both *C. plantagineum* and *H. rhodopensis* is hardly a surprise. NAC TFs regulate this wide array of physiological processes by interacting with different types of proteins, such as E3 ubiquitin ligases, phosphatases, kinases or viral proteins and binding to the promoters of downstream-regulated genes, primarily through the CGT[AG] motif [127]. Finally, the significant upregulation of sucrose synthase during dehydration is related to the accumulation of sucrose, a response that has been documented in most VDT species [38]. An intriguing finding is the abundance of transcripts encoding galactinol synthases, enzymes which catalyze the first step of the biosynthesis of raffinose family oligosaccharides (RFOs), which include raffinose, stachyose and verbascose. Normally, these metabolites serve to protect seeds from dehydration-related damage, but in this plant they occur also in vegetative tissues—predominantly as stachyose in the roots, where it may serve as a carbon store [128]. Another interesting group of transcripts induced by 48 h dehydration in *C. plantagineum* are implicated in thiamin (vitamin B1) biosynthesis and thiamin-dependent processes. This is consistent with other reports that this vitamin, besides its key role in central metabolism, is involved in adaptation to abiotic stressors like salt, cold, heat, drought and ROS accumulation [129].

Other highly represented transcripts in desiccated plants encode DNA-binding proteins, factors involved in DNA, amino acid, tryptophan, and indole-derivative metabolism, as well as cysteine proteases. The latter were previously found to be involved in programmed cell death (PCD) in plants, a genetically regulated cascade that can be triggered by severe stress stimuli [130]. Furthermore, they are involved in the degradation of storage proteins during seed development and germination [131]. Thus, the authors presume a dual role of cysteine proteases during desiccation: participation in a cellular recycling programme and/or preliminary build-up to facilitate rapid assimilation storage proteins upon rehydration [37].

Upon rehydration, the *C. plantagineum* profile is dominated by transcripts related to defense responses against stimuli of both biotic and abiotic origin. Notable examples encode enzymes responsible for ROS detoxification like peroxidases, cell wall reinforcement during pathogen attack—cinnamoyl-CoA reductase and caffeoyl-CoA O-methyltransferase, as well as chitinases and PR proteins. This suggests that the recovery of cellular homeostasis after anhydrobiosis can also be stressful to the plants and they arm themselves with the appropriate molecular tools to withstand the pressure and effectively resume growth. In addition, rehydration leads to the upregulation of transcripts involved in phylloquinone (vitamin K1) metabolism, a redox system serving as a component of the PSI complex, which may be associated with the reactivation of photosynthesis.

The RNA-seq study of *H. rhodopensis* showed that even unstressed plants have constitutively high expression of some *LEA* genes and two catalases, one of the main components of the antioxidant system. These results support the hypothesis that *H. rhodopensis* is preliminarily primed for drought/desiccation events. This is corroborated by a seemingly expanded antioxidant gene network in *H. rhodopensis*, containing more catalases, superoxide dismutases, monodehydroascorbate reductases and glutathione reductases than the desiccation sensitive *Vitis vinifera* and *Arabidopsis thaliana* [38]. Water deprivation triggered further the upregulation of enzymatic ROS scavengers, including monodehydroascorbate reductases, glutathione reductases, glutathione peroxidases, and cys-peroxiredoxins. A notable difference from *C. plantagineum* is that galactinol synthases, together with a stachyose synthase transcript, are induced only after water loss, which demonstrates that RFO accumulation in *H. rhodopensis* is a drought-responsive process. The upregulation of numerous regulatory molecules in *H. rhodopensis* during the dehydration and desiccation time points reflects the massive reorganization of the cellular machinery in these conditions. Indeed, the most abundantly induced gene encodes a putative protein phosphatase/hydrolase, suggesting that modulation of the phosphorylome is a key component of the signaling events accompanying VDT.

Members of other TF families, including the plant-specific WRKY and GRAS, and the ubiquitous MYB and HSF (heat shock transcription factors), are also affected. TFs from all these families have been shown to control various aspects of stress responses in different plant species [132–134]. Genes encoding members of an additional TF family—MADS box, and other regulatory proteins like DREB2 and NF-YA, were exclusively expressed in the water-deficient *H. rhodopensis* samples. The same transcription profile is displayed by two genes for early light-inducible proteins (ELIPs).

A distinctive feature reported for the transcriptomes of *H. rhodopensis* and *C. plantagineum* was the high amount of unknown sequences, which may be indicative for enrichment of taxonomically restricted genes (TRGs) [135]. Expression of several TRGs is modulated during the dehydration/desiccation conditions in both plants. These are of particular interest because they may reveal species-specific mechanisms contributing to VDT and throw light on the evolution of this trait. Two of the TRGs in *C. plantagineum*, encoding a cysteine-rich rehydration-responsive protein 1 (CpCRP1) and an early dehydration-responsive protein 1 (CpEDR1), respectively, have been characterized in more detail [136]. They appear to be confined to the Linderniaceae family and thus may have evolved recently. CpCRP1 supposedly facilitates the osmotic stress adjustment at the cell wall and may exert a role during rehydration. In turn, CpEDR1 exerts a protective effect on chloroplasts where it accumulates upon desiccation. Furthermore, several of the unknown transcripts are assigned to be non-coding RNAs (ncRNAs), which can control gene expression at the epigenetic, transcriptional and posttranscriptional level and thus may contribute to the molecular reprograming triggered by water loss. For example, transcript 28,852 from *C. plantagineum* which is upregulated during dehydration, though its exact function is still not determined [136].

A recent study on *H. rhodopensis* expanded our understanding of the dynamic transcriptional regulation by adding new time points during the drying curve: slight (~ 65–75% RWC, designated as D75) and severe dehydration (~ 20–25% RWC, D20) [104]. The results indicate that even slight dehydration already repressed metabolic pathways and growth/development processes and activated transcripts implicated in redox homeostasis, regulation of seed germination and responses to stimuli. On the other hand, a large portion of the accumulating transcripts during severe dehydration match the biological processes influenced by complete desiccation but have opposing expression patterns in D75 or are not detected in the moderately dehydrated plants (RWC ~ 50%) investigated in the earlier experiments [120]. These findings support the hypothesis that D20 is a crucial stage in the transcriptional regulation of VDT in *H. rhodopensis*, and further highlight the differentiation between the dehydration (moderate RWC) and desiccation (low RWC) responses which have been seen in other species [137]. The VDT-related roles of genes representative for cyclic electron flow, carbon turnover, phenylpropanoid metabolism, stress hormone signal transduction, protein quality control and DNA repair are also discussed in detail in this paper [104].

A very intriguing feature of *H. rhodopensis* is that it not only displays strong VDT capabilities but has also remarkable tolerance to other kinds of abiotic stress: high levels of oxidative stress, freezing temperatures during the winter in its mountainous habitats and even prolonged darkness without visible senescence symptoms [21, 120]. The transcriptomic responses in some of these conditions have been studied by RNA-seq. Durgud et al. [21] analyzed plants after 7- and 30-days dark treatment, as well as their recovery after transfer for 7 days back to the light. They found that darkness resulted in the strong downregulation of genes associated with photosynthesis and photorespiration, while genes for autophagy were induced. Moreover, the expression of kinases related to *SnRK1* (sucrose non-fermenting-related kinase 1), a central regulator of the reactions to stress-induced sugar and energy starvation, was also elevated. This suggests that the SnRK1 pathway was activated, which is concomitant with the available data in the literature. In an optimal environment SnRK1 activity is repressed, but it is switched on in growth-limiting conditions to promote stress responses like autophagy, protein, lipid and cell wall degradation, as well as to repress anabolic pathways [138]. Intriguingly in *H. rhodopensis*, unlike in many other species, prolonged darkness did not increase the levels of most of the chlorophyll catabolism genes and even repressed some of them, which explains the negligible chlorophyll degradation and the stay-green phenotype of the treated plants [21].

##### Studies in other Linderniacae and Gesneriaceae species

Other representatives of the genus Craterostigma as well as desert-dwelling species in the genus Lindernia also manifest VDT. A peculiar exception is *Lindernia brevidens*, which inhabits tropical rainforest regions in eastern Africa and is desiccation-tolerant despite never being subjected to prolonged drought periods in its natural habitat [8]. The VDT trait was likely inherited from desert-dwelling ancestors of *L. brevidens* before populating the rainforest environment and has been maintained in the population.

An intriguing study utilized a comparative systems biology approach between *L. brevidens* and the closely related, desiccation sensitive *Lindernia subracemosa* [13]. The transcriptomes of the two species were analyzed at control, mild dehydration (water withdrawal for 3 days), severe dehydration (drought for 7 days), desiccation (for 10 and 14 days, respectively), as well as recovery of the plants after 24 and 48 h. The results indicate that the largest degree of reprogramming occurred during the transitions from mild to severe dehydration and from desiccation to rehydration. In *L. brevidens*, the shift in gene expression patterns from severe dehydration to desiccation were relatively stable. In contrast, the responses in *L. subracemosa* were the opposite, discriminating between the high tolerance of the first species and the susceptibility and subsequent death of the second. Indeed, despite having similar transcriptomic footprints at the rehydration stages, *L. brevidens* samples eventually recovered while those of *L. subracemosa* did not. This suggests that the protective mechanisms induced during desiccation in *L. brevidens*, and which are absent in *L. subracemosa*, are important for the survival after desiccation.

The promoters of the desiccation-associated genes in *L. brevidens* are enriched for cis-regulatory elements (CREs) related to the ABA-mediated dehydration response as well as seed-maturation CREs recognized by TFs like bZIP3, AREB3 and ABI5. Overall, a wide variety of seed-specific genes were modulated only in *L. brevidens* during the stress treatment. For example, the genes encoding the seed 2S and 12S storage proteins were upregulated, while the potent controller of dormancy DELAY OF GERMINATION 1 (DOG1) was downregulated. *L. brevidens* not only possesses many more ELIP genes in the genome than *L. subracemosa*, but nearly all of them were highly expressed only during severe dehydration, desiccation, and rehydration. One third of LEA protein genes were expressed more than 30 times higher in the desiccation stage in *L. brevidens* than the respective orthologues in *L. subracemosa*, reflecting their importance for VDT. Like *C. plantagineum*, *L. brevidens* accumulates the unusual 8C sugar 2-octulose in unstressed conditions and which serves as a carbon reservoir during a water loss. The genes for transketolase enzymes involved in the production of octulose-8-phosphate were highly expressed in the controls and rehydrated samples in *L. brevidens* and *C. plantagineum* but not in *L. subracemosa*. The differences between the two closely related species support the hypothesis that acquisition of VDT is not caused by a master regulatory switch, but rather by a massive network rewiring due to the gradual accumulation of additive adaptations [13]. This is in accordance with the observed gradient of VDT phenotypes, which are more strongly pronounced in some species (*H. rhodopensis*, *C. plantagineum*) than in others (*L. brevidens*) and depend on the environmental factors [139].

A good illustration of the connection between VDT phenotype strength and the ambient conditions is provided by another member of the Gesneriaceae family—*Boea hygrometrica*. This perennial plant is widespread in East Asia and is characterized by the need for acclimation by gradual water loss before displaying full tolerance to rapid desiccation. In a study by Zhu et al. [103], samples were taken from plants that were untreated, slowly dehydrated on soil (acclimation) for 5 days (80% RWC) and 14 days (< 10% RWC), rapidly air-desiccated for 2 h (80% RWC) and 48 h (< 10% RWC), rehydrated after acclimation (3 days) and air-desiccated for 2 h (80% RWC) and 48 h (< 10% RWC) after acclimation [103]. One of the main findings in this study is that ascorbic acid biosynthesis, degradation of cell wall macromolecules, respiration and protein quality control were differentially regulated between the quickly and slowly dehydrated non-acclimated plants. Since only the second group can survive after rehydration, the results highlight the importance of these processes for cell viability during desiccation. Many of these transcripts were significantly upregulated even during the subsequent recovery phase, supporting their role as primers for the acquisition of tolerance to rapid drying. Ascorbate is among the principal water soluble non-enzymatic antioxidants and can prevent the accumulation of excessive ROS during stress [140]. Water withdrawal causes plasmolysis and mechanical strain on cell walls. The negative effects of this stress on the walls can be alleviated by cell wall folding which requires structural flexibility, including loosening [141]. In turn, protein quality control systems aid in the refolding or degradation of the misfolded and denatured proteins that accumulate in consequence of water deficit [142].

On the other hand, α-tocopherol biosynthesis and autophagy-related transcripts were differentially regulated between the air-dried samples—they are more prominently enhanced in the acclimated plants only. This suggests that they are essential for the proper VDT response. α-Tocopherol is one of the main fat soluble non-enzymatic antioxidants and protects membranes from excessive stress-induced damage and lipid peroxidation [140]. α-Tocopherol can physically interact with and deactivate singlet oxygen produced in the chloroplasts, one of the main ROS provoking photooxidative stress [143]. Autophagy is a crucial process for PCD, precursor recycling and protein degradation, especially under unfavourable conditions. Autophagy-associated transcripts responded to severe dehydration in *H. rhodopensis* as well [104]. It is notable that this mechanism also plays a major role in DT in the yeast *Saccharomyces cerevisiae*, which shows its universal importance for desiccation tolerance [144]. The comparisons of *H. rhodopensis* and *B. hygrometrica* transcriptomes show that despite being closely related and sharing many common responses, some unique features can also be distinguished. For example, dehydration-induced auxin signaling appears to be more specific to *H. rhodopensis*, while trehalose biosynthesis and ascorbic acid build-up are more typical for *B. hygrometrica* [104]. More information on the similarities and differences between the two species can be found in a recent dedicated article [142].

The second study which published -omics data for *B. hygrometrica* was devoted to the newly sequenced genome and general transcriptional reprogramming triggered by drought [12]. In this case, well-watered, moderately dehydrated (RWC ~ 70%) and desiccated (RWC ~ 10%) samples were analyzed. Enrichment analysis of the differentially expressed genes (DEGs) showed that some of the most influenced pathways are related to membrane maintenance and vesicle trafficking, ABA metabolism and signaling, and mRNA stability. Membranes are among the most sensitive sites for dehydration-induced damage and protection of their integrity is crucial for cell viability [145]. ABA can regulate DT and DT-reestablishment in orthodox seeds, like in *A. thaliana*, and the hormone can trigger a resurrection response in *C. plantagineum* calli [146]. In turn, the stimulation of the mRNA surveillance pathway points to the importance of aberrant and low-quality mRNA removal from the drying cells. Another finding in this study was that *B. hygrometrica* contains more LEA protein genes than *C. plantagineum* and *H. rhodopensis*—a total of 65, and 47 of these (72.3%) were modulated during dehydration [12]. The authors hypothesized that this can be explained by the longer and more severe drought periods that *B. hygrometrica* experiences in its natural habitat. Similarly, in the case of ELIPs, 13 out of 17 (76.5%) identified orthologues were considered as DEGs in these conditions, highlighting the significance of photosystem II protection. Alternative splicing (AS) was also shown to contribute to the transcriptome reprogramming of *B. hygrometrica* since a large part of the genes (7127) had two or more AS products.

Another desiccation-tolerant representative of the *Boea* genus with a reported transcriptome is the herb *Boea clarkeana* endemic to China. However, in this case the expression profiles were not analyzed in the context of water deprivation and the RNA was extracted from non-stressed leaf material taken from the wild [102]. In a similar fashion, very recently the transcriptome of *Ramonda serbica*, a fourth resurrection plant belonging to the Gesneriaceae family, was de novo assembled, but not evaluated with regard to desiccation tolerance [106].

##### Desiccation response in the woody plant Myrothamnus flabellifolia

The last dicotyledonous plant with a sequenced transcriptome to date is *Myrothamnus flabellifolia*, a woody species populating regions in central and southern Africa [105]. It is a member of one of the oldest families of flowering plants and thus can be considered the most primitive angiosperm to manifest VDT. The samples for sequencing were collected based on tissue water retention—controls with 100% of the regular fresh weight, slight dehydration (90% initial fresh weight, symptoms of leaf wilting), severe dehydration (75% water retention, wilted leaves), desiccation (27%, dried state), early rehydration (6 h post irrigation, leaves partially unfolded) and late rehydration (12 h, full recovery). The observed transcriptional patterns overlap to an extent with the ones for the previously discussed species—for example water loss provoked a massive induction of defense-related pathways, ABA biosynthesis and signaling, as well as various components of the antioxidant system, LEAs, sucrose synthase, etc. [105]. However, the responses of the photosynthesis-associated genes were somewhat unique. In contrast to the data for other resurrection plants, several of the upregulated transcripts in the slightly and severely dehydrated leaves were involved in photosynthesis—mainly in structural aspects like PSII assembly, thylakoid membrane organization, and chlorophyll biosynthesis. Other components of the photosynthetic apparatus, such as subunits of the reaction centers, electron transport chain proteins, and ATP synthase, were repressed. These data demonstrate a considerable modification, but not a complete inhibition, of the photosynthetic machinery in *M. flabellifolia*, probably with the aim to limit ROS generation and the subsequent oxidative stress. Another notable finding in *M. flabellifolia* is the enrichment of DEGs participating in isopentenyl pyrophosphate (IPP) biosynthesis during the later stages of dehydration and rehydration. IPP is a central metabolite and a precursor of the diverse class of isoprenoids, containing both primary and many secondary metabolites, as well as some plant hormones (gibberelins, ABA, brassinosteroids, cytokinins), many of which have functions in defense processes [147]. The massive scale of the transcriptional reprogramming triggered by water loss in this plant is evident by the impressive amounts of modulated TFs: 295 are sensitive to dehydration, of which 53 respond even at the early stage, and 287 after rehydration. Most of the affected TFs are members of the bHLH, MYB, and WRKY families. Even more remarkable is the number protein kinases (PKs) that were modulated: 484 during dehydration, with 91 rapidly induced by the slight stress, and 468 during the recovery phase. PK-exerted phosphorylation is among the primary mechanisms of protein activity regulation. This demonstrates the complexity of the dehydration response, which integrates multiple inputs not only from the altered transcriptome but also from the proteome regulation patterns.

#### Monocots

##### Transcriptional reprogramming in members of the Poaceae

*Tripogon loliiformis*, a small grass with remarkable tolerance to various extreme conditions, was the first VDT monocot subjected to RNA-seq [109]. The analysis followed the well-established scheme discussed in the previous chapter for other species—a desiccation curve, covering time points for controls, light/moderate (in this case 60% RWC) and severe dehydration (40% RWC), desiccation (< 10% RWC) and recovery (48 h after rewatering). The authors focus their attention on cell death-related processes and revealed that water loss repressed both PCD and senescence-associated genes, but induced autophagy. It was proposed that the latter was positively regulated by a build-up of trehalose. These observations were supported by physiological experiments. The pro-survival function of autophagy, which is a common strategy employed during desiccation, may be attributed to its contribution to the maintenance of the nutritional balance by strictly controlled elimination and subsequent assimilation of certain cellular structures. A second report on *T. loliiformis* compares the VDT strategies of shoots with those of the roots [94]. Intriguingly, despite the equivalent severity of the water deficit, different responses were triggered in roots. Roots still contained high levels of trehalose, but autophagy pathways were not activated in the dehydrated samples. This was supported by the lack of autophagosomes in roots when compared to shoots. The authors suggest that since roots act as sink organs with high energy reserves even during severe stress the need for autophagy is eliminated. This is in accordance with the upregulation of *Sugars Will Eventually Be Exported Transporters* (*SWEET*) transcripts in both sites, coupled with sucrose translocation to the roots. This distinct profile of the roots’ reactions to dehydration points to their crucial relevance for survival and accentuates the need for further research of this organ in resurrection species.

*Oropetium thomaeum* is a suitable model species to study VDT due to its tiny genome size of only 245 MB [148]. Recently, its desiccation responses have been characterized by a combination of –omics approaches, including transcriptomics [107]. The expression profiles were monitored through 0, 7, 14, 21 and 30 days of water deprivation as well as 1 and 2 days after rehydration. Concomitant with a seed-derived origin for VDT, the data for *O. thomeaum* support the rewiring of seed specific pathways as the primary mechanism driving the evolution of the trait in this species. For example, the total number of *LEA* genes encoded in *O. thomeaum* is similar to that in other grasses, but unlike them nearly all of the *LEAs* in *O. thomeaum* are barely detectable in hydrated tissues and strongly upregulated in the desiccated samples. This is accompanied by the concurrent activation of TFs implicated in seed development such as homologs of *DOG1*, *ABSCISSIC ACID INSENSITIVE 3 (ABI3)* and *GEm-Related 5 (GER5)*. ABI3 was originally regarded as a seed-specific TF, but recently it was shown that it acts as a global regulator of cell fate and abotic stress signaling [149]. In turn, GER5 is an ABA and stress-responsive TF implicated in multiple stages of reproductive development, with functions ranging from the control of inflorescence architecture to seed germination [150]. Another similarity between the vegetative and seed desiccation in *O. thomeaum* is the accumulation of oil bodies, which largely disappear 48 h after rewatering. There is a parallel upregulation of oleosin encoding genes during and after drought treatment, while in the controls there was no detectable expression. Oleosins are structural proteins that cover the single-layer envelope of plant lipid droplets and stabilize them by preventing them from aggregating [151]. A similar expression pattern was seen for the *O. thomeaum* homologues of *LDAP1* (lipid droplet associated protein), whose protein product is necessary for proper biogenesis of oil bodies [152]. These subcellular structures may exert a dual role during dehydration: as is the case in seeds they could serve as a rich energy source, utilized when the conditions are favourable (i.e. during the recovery phase), and/or they may have obtained novel functions like suppression of membrane coalescence and membrane repair [107]. Concerning the rearrangement of carbohydrate metabolism, the processes in *O. thomeaum* resemble those in some of the discussed dicot species. Desiccation provokes massive accumulation of RFO compounds, which is consistent with the induction of all three copies of galactinol synthase, two copies of raffinose synthase and one of stachyose synthase, as well as many other low-molecular weight sugars such as glucose, sucrose, fructose, and arabinose. Comparably to *T. loliiformis,* the transcripts for several SWEET transporters, facilitating the exchange and delivery of carbohydrates, are also highly expressed. The most probable purposes of all these saccharides are osmoprotection and energy storage, which is the same as in developing seeds. Overall, the -omics analyses of *O. thomeaum* suggest that the differential utilization of the already existing gene pool was instrumental for the VDT acquisition in this angiosperm plant. A second, chromosome-scale genome assembly was published for this species in 2018, which included 2799 newly-annotated genes that showed differential expression during desiccation/rehydration [49].

Like *O. thomaeum*, *Sporobolus stapfianus* is another C4 resurrection grass with a sequenced transcriptome. This makes them especially useful models to study, considering the enormous economic significance of other C4 members of the Poaceae family-like maize, sorghum, sugarcane and millet. The transcriptional reprogramming of *S. stapfianus* was analyzed in a dehydration/rehydration time-course, which included the well-watered controls; 4 dehydration points—80%, 60%, 40% and 30% RWC, respectively; desiccated samples with ~ 11% RWC; and 2 rehydration points of 12 and 24 h [108]. The period of peak expression for up-regulated genes and lowest expression for down-regulated genes was 30% RWC, suggesting that this water content may delineate the dehydration and desiccation response in this species. Almost half of the 50 most abundantly upregulated genes in this condition encode LEA proteins, which is not surprising given their importance for VDT. In contrast, 28 of the 50 most abundantly repressed genes were annotated as phosphoenolpyruvate carboxylases (PEPC). This is a central enzyme in C4 plants, responsible for the fixation of inorganic carbon in the bundle sheath cells by the addition of bicarbonate to phosphoenolpyruvate. Further crucial enzymes taking part in photosynthesis (RuBisCO activase) and primary energy metabolism (fructose 1,6-bisphosphate, aldolase) were also downregulated, as was reported for other VDT plants. During rehydration the strongest modulation of transcripts was observed at 12 h, suggesting that at 24 h the physiological state of the cells has already started to return to normal. The genes with the highest induction in the recovery phase were enriched for processes like protein synthesis and membrane transport. Notably, those that had maximum depletion at 12 h post-rehydration, significantly overlapped with the group that was the most abundant in the 30% RWC samples, including LEAs and other defense-related proteins. This result shows the inverse nature of the dehydration/rehydration responses. Finally, the integration of the transcriptomics with metabolomic data from *S. stapfianus* revealed that their highest concordance was in processes directed to cellular protection, especially the accumulation of antioxidants and carbohydrates with osmoprotectant functions, emphasizing their importance for VDT. To the contrary, little correlation between transcript and metabolite abundance was found for processes that do not appear to contribute directly to cellular defense, such as amino acid metabolism [108].

The most recently studied transcriptome of a VDT Poaceae grass belongs to *Eragrostis nindensis*, which was performed in parallel with the closely related desiccation sensitive crop species *Eragrostis tef* [14]. This is the first published comparative –omics analysis of a resurrection plant with a cereal crop, both belonging to the same genus. Samples were taken for comparison at moderate dehydration (56 h, D1), severe dehydration (104 h, D2) and, for *E. nindensis*, desiccation (224 h). Rehydration was monitored at 4 time points—0 h, 12 h, 24 h and 48 h. Both *E. nindensis* and the sensitive *E. tef* up-regulated “seed specific” genes during dehydration stress. This trait appears to be a widespread one in grasses, based on the authors’ analysis of publicly available expression data for several grass species. This suggests that a much smaller subset of so-called seed-specific genes are associated specifically with VDT and, additionally, that the classification of “seed specific” may often be misleading. A good illustration is the genes encoding LEA proteins: most of the *LEA* subfamilies showed the same expression trends in *E. nindensis*, *E. tef*, and even *O. thomeaum*. However, the *LEA5* and *LEA6* subfamilies were exclusively induced following desiccation and rehydration in the two resurrection species, but not in *E. tef*. Comparable expression tendencies of some *LEAs* were found also in *L. brevidens* and *L. subracemosa*—a core set of LEAs was accumulated in both species while a specific subset correlated with desiccation tolerance [13]. Analysis of the VDT-specific seed genes in grasses and other resurrection plants might help to disentangle those genes generally involved in stress response and those specific to the VDT trait.

##### Desiccation responses in Velloziaceae species

*Xerophyta humilis* is one of two Velloziaceae resurrection plants with a recently sequenced transcriptome, which was achieved in a study designed to address the question of how seed maturation genes might be reactivated in desiccating vegetative tissues [39]. The gene expression patterns in leaves collected at five different time points during a drying curve (100%, 80%, 60%, 40% and 5% RWC) were compared to the ones in developing seeds at 3 stages of developing seeds (early seed maturation, mature green and mature dry seeds, at 6, 11 and 17 days post fertilization, respectively). The data indicate massive overlap in the transcriptomic footprints between leaves and seeds, particularly genes involved in abiotic stress response, or related to ABA signaling and the ABI3 regulon. ABI3 regulon genes are directly regulated by ABI3 and, by extension, downstream components of the LAFL network: the seed master regulatory TFs LEAFY COTYLEDON 1 (LEC1), ABSCISIC ACID INSENSITIVE 3 (ABI3), FUSCA 3 (FUS3) and LEAFY COTYLEDON 2 (LEC2) [153]. However, when the behavior of the members of the LAFL network was scrutinized, it became clear that they are active only in maturing seeds and are not differentially expressed in the desiccating leaves. Another master regulator in seeds, exerting a crucial ABA and abiotic stress-modulated function during germination and seedling growth downstream of ABI3, is the bZIP transcription factor ABI5 [154]. However, similarly to the LAFL network genes, ABI5 was only induced in seeds. Thus, it appears that at least in *X. humilis*, the VDT programme is orchestrated by an alternative, LAFL-independent mechanism. The examination of the promoters of ABI3-regulon genes showed that they were depleted of the important seed RY *cis*-regulatory motif but were enriched in ABA-responsive elements (ABRE). This leads to the hypothesis that the desiccation-induced activation of the ABI3 regulon in *X. humilis* may be achieved by ABRE-binding transcription factors (ABFs), of which several were reported to be up-regulated during desiccation. Overall, in accordance with the recent data from *E. nindensis* discussed above, this study on *X. humilis* provides evidence that seed maturation and VDT indeed share common mechanisms, but the acquisition of VDT also requires additional strategies which may be unique for VDT and could, for example, involve epigenetic changes and non-coding RNAs. An interesting finding in the *X. humilis* study is a conserved duplication of ABI3 in both investigated Xerophyta species, resulting in one full-length ABI3 gene and three truncated variants lacking the B3 DNA-binding domain. While expression of the full-length ABI3 was restricted to seeds, a truncated version was induced during desiccation in leaves. The function of this gene remains unclear, but it may represent a lineage-specific adaptation relevant to VDT given that no similar duplication has been observed in any other plant species.

The genome of another *Xerophyta* species, *X. schlechteri*, was published in 2017, together with transcriptome data from several different conditions. This work has taken into consideration that briefly after germination *X. schlechteri* has increased sensitivity to rapid desiccation, but that recovery can be accelerated in seedling shoots by treatment with ABA. Therefore, the authors compared the responses of adult plants to a desiccation treatment, with sampling at 2.5 (fully hydrated), 2, 1.5, 1, 0.5 and 0.1 gH_2_O g^−1^ dwt (dry weight) of total water fraction, plus 12 h and 24 h post rehydration, with the response in shoots and roots of seedlings recovered after drying, with or without ABA treatment [25]. The expression patterns in shoots and roots of seedlings after ABA application had some common features, like upregulation of transcripts related to chlorophyll degradation and downregulation of energy metabolism, but also many distinct characteristics reflecting the different ABA sensitivity of the two organs. The co-expression networks in the ABA-treated seedlings overlapped to a large extent with those in the dehydrating mature leaves, pointing to the resemblance of the DT strategies in these two developmental stages. Intriguingly, a significant part of the nodes in the desiccation co-expression network were not sensitive to exogenous ABA, providing evidence for ABA-independent regulators and pathways. As in the case with *T. lolliiformis*, pro-apoptosis and senescence-associated genes were depleted during the dehydration course, while genes antagonizing these processes and/or involved in autophagy were induced. These anti-senescence mechanisms triggered during the desiccation of *X. schlechteri* demonstrate one of the different aspects of VDT from the common drought responses.

#### Non-angiosperm tracheophytes

The research on DT features in early diverging, seedless vascular plants, like ferns and lycophytes, has grown in popularity in recent years. Their unique intermediate phylogenetic position has the potential to shed light on important details of the evolution of VDT. Up to this point, the transcriptomes of three representatives of the ferns: *Hymenoglossum cruentum*, *Hymenophyllum caudiculatum*, and *Hymenophyllum dentatum*, and three of the lycophytes: *Selaginella lepidophylla*, *Selaginella sellowii* and *Selaginella tamariscina* have been reported.

##### Characterization of VDT in ferns

*Hymenoglossum cruentum* is an epiphytic fern that populates high humidity/low-light environments. However, there are frequent changes of the humidity in this habitat and as a result *H. cruentum* can be subjected to rapid desiccation. A study including transcriptomic analysis of this plant was published in 2020 [110]. The gene expression levels were monitored in frond samples with 100, 80, 6% RWC for drying and 80% RWC for recovery, respectively. Overall, the number of DEGs across the different conditions was relatively low, suggesting that unlike angiosperms *H. cruentum* utilizes a constitutive mechanism to survive desiccation. After creating and examining self-organizing maps (SOM), the authors report 26 DEGs that potentially have key roles during the dehydration/rehydration cycle in *H. cruentum*. The co-expressed genes in the fully hydrated controls were involved in the photosynthetic apparatus, intracellular mobility, the antioxidant system, protein turnover and stabilization. However, the material used for the study was initially collected from the wild, which cannot exclude previous exposure to desiccation/rehydration resulting in stress memory and a priori influence on the expression behaviour. The genes abundantly expressed during water loss were primarily related to cell protection and stress signalling, for example an apoplastic catalase (*CAT3*), a peroxidase, dehydrins, and AP2/ERFs; as well as control of cell death (*MIEL1*). In turn, the large APETALA2/ETHYLENE RESPONSIVE FACTOR (AP2/ERF) family of plant-specific TFs contains potential mediators of stress responses, many of which are sensitive to ABA or ethylene [155]. MYB30-Interacting E3 Ligase 1 (MIEL1) is an ubiquitin-protein ligase that leads to the proteasomal degradation of the positive regulator of hypersensitive cell death MYB30 [156]. MIEL1 can thus function as an attenuator of cell death. Interestingly, an ELIP gene (ELIP9) was shown to accumulate not during the desiccation phase but after rehydration. This may reflect a high oxidative load during recovery, which turns it into a stressful period for the *H. cruentum* desiccation response. Thus, to shield itself, the plant keeps the antioxidant and photoprotection systems activated. In agreement with this, other genes with the highest expression during rehydration are two LEA transcripts, a peroxidase and a glutathione-S-transferase (GST).

Transcriptomic datasets for other two fern species—*Hymenophyllum caudiculatum* and *Hymenophyllum dentatum*, were generated for a comparative study aiming to discriminate the DT mechanisms in different microenvironments:*. caudiculatum* is restricted to the lower canopy of Chilean temperate rainforests, while *H. dentatum* occupies both the upper and lower canopies [111]. Samples from fully hydrated, dehydrated (~ 60% RWC, at the first day of water withdrawal), desiccated (at the seventh day), partially (~ 60% RWC) and fully rehydrated (~ 90% RWC) fronds were analyzed. In accordance with previous reports [157] and the results for the related *Hymenoglossum cruentum*, these two filmy ferns also had relatively few DEGs across all time points, pointing to a constitutive VDT strategy. Regardless of the hydration status of the fronds, transcripts involved in photosynthesis, translation and the antioxidant system were always abundant in both species, which probably reflects their homoiochlorophyllous nature and their hardwired state of stress-alert. The accumulation of transcripts encoding GSTs and ferritins was observed in both species during dehydration with a decrease during recovery. GSTs are multifunctional enzymes known for their ability to detoxify xenobiotic and endobiotic compounds, including ROS, through conjugation to glutathione [158]. Their upregulation is associated with the need to cope with oxidative stress and cytotoxic molecules. Notably, the observed downregulation after rehydration in *H. caudiculatum* and *H. dentatum* is the opposite of what was found in *Hymenophyllum caudiculatum*, an example of a genus-specific transcriptomic signature. Ferritins, as iron-binding proteins, have a key role in redox homeostasis and can protect against free-iron mediated oxidative stress [159]. Apart from these common mechanisms, the VDT strategies of *H. caudiculatum* and *H. dentatum* showed some distinct features, which may be due to a combination of species-specific and microhabitat-related factors. For instance, *H. caudiculatum* had almost twice as many DEGs as *H. dentatum* across the different conditions, demonstrating a more robust reprogramming. While the characteristic responses in *H. caudiculatum* concern mostly osmotic balance and phenylpropanoid pathways, in *H. dentatum* they are oriented towards the defense system and protection from high light stress. This is in line with the habitat distribution of the two plants as *H. dentatum* occupies more illuminated sites in the higher canopy of the host trees.

##### Transcriptomic studies in DT lycophytes

Lycophytes represent one of the oldest lineages of vascular plants. Only three DT lycophyte species, all from the genus *Selaginella*, have published transcriptomes. The first to be published was the one of *S. lepidophylla*, an exceptionally resilient plant that can survive in anhydrobiosis for decades [26]. In contrast to the studies discussed above, the order of the treatments was shifted—the experiment was initiated with desiccated plants (0 h), then samples were collected consecutively at 1 h, 6 h, 24 h (partial recovery) and 120 h (full recovery) after rehydration, and finally 24 h after de novo induced dehydration. Strikingly, despite storage of the plant for three years in a quiescent state before the treatments, 77% of the genes in *S. lepidophylla* had a detectable expression in desiccated leaves, suggesting very effective mechanisms to maintain the mRNA pool. During recovery, the transcription patterns were similar, with a relatively low number of DEGs. Co-expression clusters in these time points were enriched mostly for genes related to DNA repair and stress responses. In turn, the transcription profiles during desiccation resembled a large extent the ones from angiosperm resurrection plants with a high representation of genes involved in cell protection, osmoregulation and protein stabilization, including a considerable number of LEAs and ELIPs, families in *S. lepidophylla* that have dramatically expanded. However, as in the case of *H. rhodopensis*, LEAs were found to be abundant also in the well-hydrated tissues, pointing to their possible role in the constitutive defense. It must be noted that in the genome of *S. lepidophylla* many desiccation-associated genes were reported to reside in large tandem arrays characterized with similar expression patterns. This strategy provides the benefit of enabling coordinated regulation of the adjacent genes and abundant transcript accumulation in a short time span. This may have played an important role in the acquisition of DT by this plant. Coordinated expression of genes arranged in a cluster may also protect against mutations which occur in one gene of the cluster.

*S. tamariscina* is another VDT Selaginella species and its transcriptome was recently studied in parallel to one of the desiccation sensitive model plant *S. moellendorffii* [113]. However, in this case only fully hydrated and moderately dehydrated samples were sequenced (100% and 50% RWC for both plants) while desiccation/recovery time points were omitted. Overall, the authors describe 133 genes as drought responsive in *S. tamariscina* and highlight the importance of four major functional categories: antioxidant activity, osmotic balance, cuticle defense and signal transduction. Detailed kinetic expression studies indicate that their abundance was gradually increasing during water loss, reaching a peak at the last dehydration state (12 h), and then declining to their original levels during the recovery. Thus, all of them are linked to the dehydration responses of *S. tamariscina*.

The transcriptome of the third DT representative of *Selaginella*, *S. sellowii*, was compared with the desiccation tolerant *S. lepidophylla* and the desiccation sensitive *S. denticulata* [112]. *S. lepidophylla* and *S. sellowii* were selected since both manifest VDT but have different morphologies, thus providing the opportunity to identify common DT strategies that are independent of morphological adaptations. RNA was extracted from unstressed controls, two phases of partial dehydration (70% and 50% RWC) and dried material (10% RWC). In the case of S. *lepidophylla* and *S. sellowii* samples were taken also 2 h and 6 h after rehydration. The observed expression patterns were markedly different, even between the two VDT species: the shared response by orthogroups to dehydration was 26.82% and 20.5% for *S. sellowii* and *S. lepidophylla*, respectively. This shows that even in these closely related plants, VDT has probably been acquired through convergent evolution. However, subsequent evaluation of enrichment for functional categories revealed some common processes. For example, induction of transcripts associated with amino acid and secondary metabolism, redox homeostasis and transport was characteristic only for the tolerant species. In the case of amino acids, during the dehydration treatment both *S. sellowii* and *S. lepidophylla* activated more genes related to synthesis than degradation, pointing to the importance of the maintenance of the amino acid pool. The most abundant subcategories of secondary metabolism concerned phenylpropanoid and flavonoid pathways, from which many potent protective antioxidants can be derived. In turn, shared regulators of the redox balance include glutathione, ascorbate, peroxiredoxins and thioredoxins. Only *S. lepidophylla* additionally upregulated genes, encoding some other enzymatic ROS-scavengers like catalases and superoxide dismutases. With regard to transport, a similarity between *S. sellowii* and *S. lepidophylla* was the analogous expression pattern of some major intrinsic prtoteins (MIPs), transmembrane channels transferring water and small solutes across the plasma membrane. Calcium transporters were specifically modulated in *S. sellowii*, compared to only porins in *S. lepidophylla*. Other differences are related to transcripts involved in cell wall modification (in *S. lepidophylla*) and an unexpected dehydration-induced upregulation of photosynthesis-related genes in *S. sellowii*. The latter is in accordance with the faster recovery of photosynthesis during rehydration in *S. sellowii* than in *S. lepidophylla*. Altogether, it seems that these two closely related DT plants employ a different subset of genes to influence partially overlapping biological processes, with some species-specific adjustments.

### Non-vascular plants

Research on VDT in non-vascular plants is indispensable for deciphering the constitutive DT response and elucidation of the evolutionary origin of the VDT phenomenon. It may help shed light on whether similar desiccation-activated pathways in separate VDT lineages have arisen through convergent evolution or were, as hypothesized, already present in common ancestors. It will increase our knowledge on the mechanisms that allow tolerance to rapid desiccation, which is not achievable by vascular plants endowed with inducible VDT. Detailed molecular studies on DT in non-tracheophyte species is still limited, but there has been progress recently (Table 4).

#### Mosses

Moss from the genus *Syntrichia* are important models of VDT. The species *S. ruralis* (previously *Tortula ruralis*) was one of the first species with non-NGS sequencing data available. The study reported massive upregulation of LEAs, factors involved in protein synthesis, membrane formation and repair, redox homeostasis and protein phosphorylation from over 10,000 ESTs [160]. Another species, *S. caninervis*, has been analyzed more recently by RNA-seq [40, 98]. This desert plant is exposed to unpredictable and frequent instances of desiccation/rehydration during its lifecycle. In the first study, material from air-dried gametophores that were rehydrated for 24 h, as well as secondarily dried ones for 0.5, 1, 1.5, 2, 4, 6, 8, 10, 12 and 24 h, was subjected to high-throughput RNA-sequencing to assemble a global transcriptome. Annotation and GO enrichment analysis revealed abundant transcripts in categories such as response to abiotic stress, photosynthesis and membrane integrity. These were used to identify, classify and characterize the AP2/ERF gene family in *S. caninervis* [161]. The genome for this species was recently published, and included a secondary transcriptomics analysis [40]. The conditions analysed included full hydration at 20 °C, slow drying (at 67% relative humidity), rapid drying (over activated silica gel for 30 min), rehydration of slowly dried samples (for 30 min), low temperature (4 °C for 90 min), elevated temperature (30 °C for 90 min) and heat shock (35 °C for 90 min). The results revealed massive transcriptomic reprogramming in all treatments except for 4 °C. Furthermore, the authors included a dominant patterns (DP) analysis to link the transcriptomic response to the genomic context. The composition of the obtained desiccation-specific DPs was characterized by the presence of ELIPs, LEAs and factors from the ABA signaling pathways, as well as E3 ubiquitin-protein ligases, suggesting an important role of protein homeostasis. An interesting feature of the rehydration-associated DPs is the increased abundance of ethylene-responsive TFs, which is indicative of the stressful nature of this condition. Finally, by incorporation of the genomic data, four gene sets were identified containing clusters of three duplicated genes: little protein 1 (SALP1), situated in the plasma membrane and potentially involved in osmoregulation; the redoxresponsive chloroplast protein rubredoxin; the Golgi apparatus membrane protein TVP15, with a role in membrane trafficking; and LRR receptor-like serine/threonine-protein kinase, which might be implicated in various stresses. In each set, the duplicated genes were closely linked on the genome and up-regulated in the same manner in desiccating tissues.

*Bryum argenteum* is a cosmopolitan species that thrives in many different environments. For example, together with *S. caninervis, B. argenteum* is the predominant moss in Chinese cold deserts and must withstand the same repetitive cycles of dehydration/desiccation recovery. Initially, the transcriptomes of dried and rehydrated (2 h and 24 h) samples were sequenced [114]. The analysis of DEGs revealed that the early responses to rehydration concern mainly adaptation to stress and maintenance of the membrane integrity. Thereafter, at 24 h, genes mainly related to photosynthesis are triggered. These processes are orchestrated by many upregulated TFs, with AP2/ERF being the best represented family among them. The observed expression patterns suggest that the renewed intake of water presents a stress challenge, and thus appropriate protection mechanisms are activated first and only then the complete restoration of photosynthesis is allowed.

A second, optimized transcriptome-based study of *B. argenteum* was published in 2017 [115]. This time, fully-hydrated gametophores served as controls and four additional treatments were applied: dehydration for 2 h and 24 h, and rehydration for 2 h and 48 h. The data confirmed that early rehydration induces the most stress-associated changes in gene expression in *B. argenteum*. For instance, there was a marked accumulation of heat shock proteins (HSPs) like the HSP70 and HSP90 families. HSPs are ubiquitous molecular chaperons whose main role is to ensure proper folding and stabilize proteins especially under stress [162]. In higher VDT plants, such as *H. rhodopensis* and *O. thomeaum*, an increase of HSPs was more typical for the dehydration conditions [107, 120]. In contrast to 2 h post-rehydration, 48 h seemed sufficient for functional recovery as evidenced by the high abundance of transcripts involved in photosynthesis, translation, cell cycle and even reproduction at this time point. Notably, a large part of the cell cycle and reproduction were repressed during the early phase, reflecting the rapid transition from the state of alert to normal growth and positive carbon balance. Considering the dehydration treatment, genes related to starch and sucrose metabolism were among the most significantly altered, including a drastically upregulated sucrose synthase, as was the case with the dicot *H. rhodopensis*. This is in accordance with the powerful protectant properties that sucrose and other sugars exert during drying. Likewise, factors engaging in repair mechanisms accumulated fast but were maintained throughout desiccation. Two other interesting transcripts, encoding 9-cis-epoxycarotenoid dioxygenase (NCED), the rate-limiting enzyme for ABA biosynthesis, were induced upon dehydration and depleted upon full recovery, implying an important role of ABA for DT in *B. argenteum*. A similar expression profile had several phospholipases D (PLDs). Previously, these enzymes were shown to be activated within minutes of water withdrawal in *C. plantagineum* [163]. Their function is to produce secondary messengers that can relay and amplify a stress signal. These examples demonstrate that some of the responses of the moss *B. argenteum*, characterized with a constitutive VDT strategy, resemble the ones of angiosperm resurrection plants. Similar to *S. caninervis*, the transcriptome dataset of *B. argenteum* was later used for a detailed investigation on the AP2/ERF family of TFs, including some functional analyses [164]*.*

The model organism *Physcomitrium* (*Physcomitrella*) *patens* displays VDT only after priming with ABA [165]. The DT response is mediated by *ABI3* and a mutation in this gene results in the loss of tolerance [166]. The popularity of *P. patens* as a model organism is one of the reasons for the publication of multiple RNA-seq datasets in the last 10 years, many of which are related to development—but some also consisting of hormone and stress treatments. For instance, in a study devoted to the *ABA NON-RESPONSIVE* (*ANR*) gene, Stevenson et al. [92] performed a global transcriptional profiling of wild-type and mutant chloronemata treated with ABA, osmotic stress or dehydration (70% RWC). Intriguingly, the wild type was characterized with largely overlapping responses to these three conditions when compared to controls, including massive induction of transcripts involved in protection and repression of components of the secondary metabolism machinery, especially the phenylpropanoid pathway.

Later, a *P. patens* gene atlas containing 99 independent libraries spanning 34 different developmental stages and conditions, including dehydration and rehydration, was published [116]. For dehydration, gametophores were exposed to dry atmosphere until constant weight was reached, which corresponded to − 13 MPa water potential, and sampled 30 h thereafter. For rehydration, the material was collected two hours after recovery. Notably, only 10 DEGs were reported for these two experiments, which may reflect the relatively short time window between them. Thus, the additional analysis should focus on prolonging the period after rehydration. Considering the ABA-dependence of DT in *P. patens*, a future detailed comparison of multiple-point dehydration curves in the same tissues, treated or not with ABA, will be valuable because it will throw light on specific features of the molecular mechanisms of drought and desiccation tolerance.

#### Green algae

The first study reporting the transcriptome of a streptophyte green alga subjected to desiccation (< 10% RWC), is from 2014 and the target organism was the alpine aeroterrestrial species *Klebsormidium crenulatum* which is an early-branching member of the sister lineage to land plants [167] and thus interesting to investigate in the context of plant adaptation to terrestrial environments, including acquisition of the DT trait [117]. The assembly of the transcriptome revealed that ~ 7000 contigs were modulated after rapid desiccation, with a prevailing number of downregulated ones. The experiment, however, did not include sequencing of transcripts from cells recovered after rehydration. Most of the abundantly desiccation-induced genes do not have known homologues in green plants and a significant portion were unique, suggesting specific adaptations of *K. crenulatum* to its environment. From the remaining upregulated contigs, three major categories could be discriminated after KEGG-pathway enrichment analysis: energy metabolism, photosynthesis, and protection against light damage and oxidative stress. The upregulation of genes involved in photosynthesis is intriguing, since the desiccation treatment resulted in the shutdown of this process, suggesting that the observed transcriptional reprogramming is part of a strategy to prepare for recovery. The activation of energy metabolism pathways like the TCA cycle, glycolysis and respiration, is in accordance with the inhibition of photosynthesis—these can serve as the primary sources of ATP in the stressed cells. Integrative cellular functions such as cell division, amino acid biosynthesis, and somewhat counterintuitively—DNA repair, appeared to be significantly suppressed. Overall, the careful examination of some of the most modulated transcripts in this study revealed that a considerable fraction of *K. crenulatum* desiccation response is similar to those seen in embryophytes. This is consistent with the hypothesis that complex land plants inherited the core DT strategies from their streptophyte ancestors. These common tactics include increasing the abundance of LEAs, ROS scavengers (catalase, enzymes involved in glutathione metabolism) and factors contributing to the accumulation of compatible solutes (sucrose synthase, galactinol synthase and other enzymes leading to the production of RFOs, carbohydrate transporters). One of the most abundantly induced contigs in *K. crenulatum* (~ 36-fold) encodes an ELIP, which is another typical reaction in angiosperm resurrection plants. Interestingly, while no homologues of the auxin and gibberellin signaling cascade were discovered in the transcriptome of *K. crenulatum*, other phytohormones were well represented, for example components of the cytokinin and ABA pathways. This demonstrates that some crucial phytohormone functions were probably already established early in the evolution of plants and this could have played a key role in the transition to terrestrial habitats [168].

Chlorophyta, a sister clade of green algae to Streptophyta, is rich in species that have a land-based lifestyle. Many of them have adopted symbiotic strategies, one of the most widespread and successful of which is lichenization. In their terrestrial habitats, lichens are often subjected to severe water shortage, but quickly resume growth upon rehydration. *Trebouxia gelatinosa* is a member of the most common lichen-forming genus, in which all representatives are DT, and these features are exhibited both in lichen symbionts and in free-living individuals. The transcriptional reprogramming of the species was studied in unstressed controls, 10 h after dehydration and 12 h after rehydration [118]. The majority of transcripts (91.83%) displayed a stable expression pattern, which is consistent with a predominantly constitutive component of the VDT programme. Regarding the less pronounced inducible mechanisms, some are common among DT species, while others seem specific for *T. gelatinosa*. For instance, the behaviour of photosynthesis-related genes resembles *K. crenulatum* and their upregulation in response to water loss is probably one of the reasons for the very fast recovery of photosynthetic activity upon rehydration. Several aquaporins were also significantly induced, which may lead to increased membrane permeability and transport processes. This can be interpreted as an adaptation to mitigate the membrane damage during the rapid water influx upon rehydration. Most elements of the antioxidant system in *T. gelatinosa* were not modulated in this experiment, reflecting the constitutive aspect of the DT trait. One of the notable exceptions is the accumulation of two dihydroflavonol-4-reductases (DFRs). This enzyme catalyzes a crucial step in the biosynthesis of anthocyanins—secondary metabolites with well documented ROS-scavenging properties [169]. Interestingly, only three *LEA* genes were detected in this alga and their expression was not dependent on the hydration level. This contrasts with most other DT species, in which the *LEA* gene family is expanded and well responsive to dehydration and suggests that *T. gelatinosa* relies on alternative DT strategies. An example thereof is the observed diversification of the desiccation-related protein (DRP) family, amounting to 13 representatives in *T. gelatinosa*, 9 of which were modulated in at least one of the two treatments. The DRPs mechanism of action and contribution to DT is still unknown, but the presence of a ferritin-like domain, characteristic of other families implicated in stress protection, supports their involvement in cell defense. An important finding is that *T. gelatinosa* DRPs have higher homology to those present in extremophile bacteria than in higher plants, which supports a bacterial origin of the *T. gelatinosa* DRPs through horizontal gene transfer [118].

A third transcriptomic study of a DT alga was focused on the streptophyte *Zygnema circumcarinatum* [119]. This species is a member of the sister clade of land plants [170], but it is not early-branching like *K. crenulatum*, therefore, unraveling its desiccation responses is important from an evolutionary perspective. In *Z. circumcarinatum* the DT phenotype is dependent on the physiological state of the cells, with more mature cells being more tolerant to stresses. To understand the effects of these different levels of hardening on the molecular reprogramming caused by water deprivation, the authors analyzed material from algal cultures, either grown in liquid medium for 1 month or on agar plates for 7 months (more mature), subjected or not to desiccation. The results indicate that the algae cultivated in liquid had a much more pronounced modulation of global expression, which suggests that the population on agar was pre-acclimated to dehydration. This is supported also by the behavior of some transcripts involved in defense against ROS: glutathione-*S*-transferase, peroxisomal catalase, peroxiredoxin and peptide methionine sulfoxide reductase were all induced, but much more strongly in the agar plate variant. Similarly, photosynthesis-related genes were mostly repressed after dehydration, with a more pronounced effect in the liquid culture, as would be expected for non-acclimated cells. Intriguingly, this adaptation of the photosynthetic apparatus resembles one of some higher plants like *H. rhodopensis* and *C. plantagineum* [37, 120], but is the opposite to the responses in the other investigated streptophyte, *K. crenulatum* [117]. This reflects on one hand the convergence of the VDT strategies and on the other hand the ability of even related organisms to adopt alternative approaches to achieve DT. Many of the remaining induced transcripts in *Z. circumcarinatum* in both culture conditions were described also in other DT plants and encode enzymes involved in sucrose metabolism, carbohydrate transporters, aquaporins, ELIPs, LEAs and other chaperones, DNA repair proteins, etc. In both the liquid and agar plate cultures, however, the total number of DEGs was low, again pointing to the prevalence of constitutively acting mechanisms, like in the other non-vascular DT species.

A very recent publication is dedicated to comparative transcriptome analysis of three Chlorophyta species belonging to the family of Scenedesmaceae [100]. These include the DT algae *Acutodesmus deserticola* and *Flechtneria rotunda*, which inhabit desert microbiotic crusts and can survive multiple drying/rehydration cycles throughout their lifespan, and the aquatic desiccation sensitive relative *Enallax costatus*. Samples were taken from hydrated controls, early and late dehydration (2.5 and 7.5 h, corresponding to an algal dot volume loss of 25% and 60%, respectively), fully dried stage and recovery after 1 h rehydration. The induced and suppressed DEGs in all three species had quite distinct expression patterns: while downregulation was observed to occur at a slower, but gradual rate throughout the entire treatment, most upregulated genes remained relatively stable until the late dehydration point, after which a sharp peak in their abundance was detected. Even though the experiment was performed in the dark, photosystems repair and high-light stress-related transcripts were among the upregulated in *A. deserticola* and *F. rotunda* and light harvesting-associated transcripts in *E. costatus*. However, the main difference between the desert taxa and the aquatic one was that no specific biological process was negatively influenced in *E. costatus*. In fact, many of the suppressed processes in *A. deserticola* and *F. rotunda* were induced in *E. costatus*. Considering that some of these are involved in central metabolism, it can be speculated that a general deceleration of metabolic activity occurs only in the DT algae, but this is not characteristic for the sensitive counterpart. Another feature shared only by *A. deserticola* and *F. rotunda* was the increase of representatives of five stress-responsive TF families: MYB, B3, TRAF, GNAT and CSD, which might have a certain contribution for their survival. On the other hand, the upregulation of some well-described markers for DT, mostly involved in cell defense, like ELIPs and LEAs, was common for the three species. These findings support the hypothesis that the induction of DT-associated protective molecules is a necessary but not sufficient prerequisite to manifest DT and that it needs to be coupled to a metabolic slowdown.

## Summary of the transcriptomic data and future perspectives

The number of available transcriptomic studies on desiccation-tolerant plant species has significantly increased over the last few years. The aim of this chapter was to provide an overview of the main findings. The different species, respective reference research papers and the conditions in which the material was sampled, are summarized in Table 4. The available data show that there are several universally utilized strategies across all VDT taxa, mostly regarding activation of cell protection mechanisms like accumulation of LEAs, ELIPs, HSPs, compatible solutes and antioxidants. These, however, seem to be insufficient to confer DT by themselves and need to be complemented by additional species-specific responses, which have unique features even in closely related organisms (e.g., *S. sellowii* and *S. lepidophylla*). The still limited number of comparative analyses with non-DT species suggests that some of the commonly regulated genes may have a broader function in the context of drought/dehydration. Further comparative studies that include sensitive plants will be needed in the future to better delineate the factors required for the transition from non-DT to DT phenotype. Another question that can be further clarified is in which conditions accumulated transcripts in response to dehydration/desiccation are translated? For example, what proportion of transcribed genes are translated during desiccation or stored and translated during recovery. For this purpose, transcriptomics and translatomics data should be integrated. As noted by Oliver et al. [1], another problem that currently complicates extracting meaningful biological information from different experiments is the inconsistency in the monitoring of water content [1]. Thus, it will be recommendable that standards in the measurement and reporting of this crucial physiological parameter are widely accepted and adopted by the research community working on this topic. A similar agreement about the processing of RNA-seq outputs and the criteria to define differential expression will also improve and facilitate data mining. In a very recent publication, Marks et al. [139] discuss in detail another of the present challenges—the serious degree of variability of the plant material, originating from sampling plants cultivated in the laboratory in certain studies vs. collected from the field in others, differences between populations of the same species, and even between parts and developmental stages of the same organism [139]. This can be addressed by increasing the scope of future experiments to account for such sources of variation and has the potential to reveal critical aspects of DT acquisition and plasticity. Finally, an important step towards achieving practical applications in agriculturally relevant crops will be to conduct functional analysis of candidate DT-related genes identified by the transcriptomics approach in tolerant plants.

## Proteomics studies on desiccation-tolerant plants

VDT is associated with widespread changes in protein abundance, subcellular localisation, and post-translational modifications. However, there have been very few published proteomics studies from resurrection species in the last decade despite the surge in the generation of genomic, transcriptomic and metabolomic data. Prior to this, proteomics studies had also not been widely performed and only a limited range of proteins had been analysed across various species [121, 171].

Amongst the angiosperms, proteomics data has been published for *X. viscosa* [172, 173], *B. hygrometrica* [174], *S. stapfianus* [175], *H. rhodopensis* [176] and *C. plantagineum* [88]. Of the non-angiosperm species, proteomics datasets are available for the moss *P. patens* [177, 178], the Selaginella spikemosses *S. bryopteris* [179] and *S. tamscarina* [180] and the green alga *Asterochloris erici* [181]. The existing studies largely confirm the observations and predictions made from transcriptomic data [121, 182–184]. Unsurprisingly, many proteins show specific patterns of expression in drying tissue or display altered abundance throughout the dehydration-rehydration cycle. Of the known proteins identified, most were involved in photosynthesis, energy metabolism or stress response.

A recent combined transcriptomic, metabolomic and proteomics analysis of dehydrating *C. plantagineum* provides a more detailed snapshot of protein changes during desiccation [88]. The authors identified over 1400 proteins, of which 43% showed significant changes in abundance during VDT. Interestingly, most proteins appeared to have a low correlation between their abundance and the expression levels of the associated mRNA transcript. This suggests extensive utilisation of post-transcriptional/translational regulation during VDT, something that has been observed in seeds and other plant tissues under stress and that has been suggested to occur in resurrection plants [1, 5, 185]. This study reinforces the need for a systems-based methodology to disentangle such instances where the results of a single approach are incomplete.

## Metabolome reconfigurations in resurrection plants during desiccation

The transcript changes in response to desiccation and rehydration have been described in detail above, but metabolites also play a key role in the acquisition of VDT. De novo synthesis and increase or decrease in degradation of metabolites are modulated by activities of enzymes, which are in turn determined by gene expression. Although the number of metabolome studies has been increasing, there is much less data available compared to transcriptomic ones.

Plants alter their metabolomes in response to various stresses, including desiccation [38]. While some of the changes in metabolite abundance are merely a consequence of the encountered drought stress, others are important for the acquisition of desiccation tolerance. A growing number of metabolome studies have revealed a few metabolites that accumulate in almost all resurrection species and are therefore likely to have a general role in desiccation tolerance, as well as metabolites which are specific for certain resurrection species. Overall, the metabolomes of the resurrection species change dramatically during desiccation as well as after subsequent rehydration, to meet the challenges the plant cell is facing during these extreme conditions. Here we describe these metabolome reconfigurations and link them to the transcriptome and proteome changes to explain the desiccation tolerance in a systemic manner.

### Primary metabolites and desiccation tolerance

Sucrose is a metabolite that accumulates in all investigated resurrection species during desiccation [15, 16, 108, 120, 186]. It can already be present at very high concentrations in resurrection species even at non-stressful conditions. The desiccation-tolerant grass *Sporobolus stapfianus* contains more sucrose than the desiccation-sensitive *Sporobolus pyramidalis* [15]. Other intrinsic differences between the two Sporobolus species include higher levels of glucose and fructose but lower levels of several energy-related metabolites from the glycolysis and the TCA cycle in *S. stapfianus* [15]. The desiccation tolerant *H. rhodopensis* has much more sucrose than the desiccation sensitive species *A. thaliana* and *Thellungiella halophyla* even in the absence of stress, as well as higher levels of other sugars, polyols, and organic acids such as raffinose, melibiose, trehalose, rhamnose, myo-inositol, sorbitol, galactinol, erythronate, threonate, 2-oxoglutarate, citrate, and glycerol [120]. Replacement of water by sucrose has been suggested as the mechanism that prevents intramolecular aggregation, protein denaturation, and cellular damage that may occur when the cellular water content becomes critically low [187]. The high sucrose content is hypothesized to maintain membrane integrity and biochemical reactions, which can occur even at very low water contents [188]. One possible explanation is the formation of natural deep eutectic solvents (NaDES) by sucrose and other metabolites (organic acids, amino acids), which would lead to a liquid state that would allow biochemical processes and gene transcription to occur even in the absence of water as solvent.

Concomitant with the increase in sucrose during desiccation, starch is metabolized, which is evident also by the increased maltose levels—a product of starch degradation—and the induction of amylase genes [120]. The starch degradation may serve two purposes: providing energy and glucose moieties for the synthesis of sucrose and polysaccharides during drought and desiccation. In addition to sucrose, the raffinose family oligosaccharides stachyose and verbascose also increase during desiccation, which have been speculated to exert a protective effect against the dehydration-induced oxidative stress [120, 189].

The desiccation-tolerant *C. plantagineum* possesses high amounts of the unusual monosaccharide octulose, which is found in a few other plants as well [190, 191]. The desiccation-tolerant *L. brevidens* has more octulose than the sensitive *L. subracemosa* and a positive correlation between octulose and desiccation tolerance was shown within the Linderniaceae [191]. Octulose is hypothesized to play a role in carbohydrate storage and ROS scavenging [191]. During desiccation, when photosynthesis is impaired, carbohydrate reserves such as starch and octulose become especially important not only as an energy source but also for the synthesis of protective sugars (sucrose, raffinose, stachyose), antioxidants, secondary metabolites (anthocyanins, etc.), and amino acids [192].

Several amino acids also increase during dehydration [120, 175, 189]. In particular, gamma aminobutyric acid (GABA) seems to accumulate in many resurrection species that undergo desiccation, including *Barbacenia purpurea* and *Haberlea rhodopensis* [120, 189]. GABA is a stress metabolite which accumulates under various stresses and may control growth. Cessation of growth is important during drought stress and high concentrations of GABA in plants can redirect resources from growth to synthesis of stress-protective proteins and metabolites [193]. In turn, asparagine increases in the desiccation tolerant *Barbacenia purpurea* [189]. This amino acid is over 450-fold more abundant in the desiccation tolerant *S. stapfianus* than the desiccation-sensitive *S. pyramidalis* [15].

### Secondary metabolites in desiccation-tolerant plants

Resurrection species synthesize a variety of secondary metabolites, some of which are highly abundant during desiccation. The leaves of *M. flabellifolia* are rich in polyphenols. The predominant polyphenol in this species is 3,4,5 tri-*O*-galloylquinic acid, present at 44% (by weight) in hydrated and 74% (by weight) in dehydrated leaves [18]. The resurrection plant *B. purpurea* accumulates caffeoyl-quinic acid, which has strong antioxidant activity [189]. Metabolic profiling indicates that the composition of flavonoids can vary depending on the aridity of the regions from where the plants were collected [194]. Metabolomics has even been suggested as an approach to identify populations of *M. flabellifolia* collected from different geographical regions [195].

Metabolic profiling of *H. rhodopensis* revealed that its leaves contain high amounts of phenolic compounds and the glycoside myconoside [196]. Studies in vitro indicated that the myconoside has the highest antioxidant properties among the evaluated phenolic compounds and may thus contribute to desiccation tolerance.

Different phenylpropanoid glycosides were characterised in *C. plantagineum*, *L. brevidens*, and *L. subracemosa* and the analysis showed that verbascoside represents the most abundant phenolic compound in the three species. The amount of verbascoside correlates with desiccation tolerance and the profiles of the phenolic compounds are similar in the desiccation-tolerant species *C. plantagineum* and *L. brevidens* but differed in the desiccation sensitive *L. subracemosa* [197]. It is speculated that verbascoside contributes to oxidative stress relief during desiccation.

Anthocyanins accumulate during desiccation in many other species, which is often discernible as a visible change in color upon drying [187]. Measuring anthocyanin abundances confirm these observations [186, 198]. In addition, many unnamed secondary metabolites have been identified in resurrection plants [16, 120]. Some of them increase dramatically during desiccation, indicating a possible role in the protection against drought stress. For example, fourteen unnamed compounds have accumulated considerably in the resurrection plant *S. lepidophylla* but not in its desiccation sensitive relative *S. moellendorffii* [16]. Likewise, a number of unknown secondary metabolites were changed significantly during desiccation and others - during rehydration in *H. rhodopensis*, indicating the importance of metabolome reconfiguration during rehydration as well [120].

The available studies on secondary metabolites in desiccation-tolerant plants show that species-specific metabolites are often synthesized and they may reflect an adaptation to the particular habitat. However, a cautious analysis may suggest that many of these metabolites have an antioxidative stress potential.

### Desiccation tolerant plants synthesize and accumulate secondary metabolites with potential medical applications

Secondary metabolites from resurrection plants can have biotechnological applications and use in medicine and cosmetics [199]. The shrub *M. flabelifolia* is used extensively in ethnopharmacology against inflammation and viral diseases [200–202]. Studies in vitro show that the 3,4,5 tri-*O*-galloylquinic acid from *M flabelifolia* protects membranes against desiccation and free radical-induced oxidation [18]. Furthermore, 3,4,5 tri-*O*-galloylquinic acid inhibits the HIV and MMLV reverse transcriptases [19].

The myconoside from *H. rhodopensis* induces the nuclear factor (erythroid-derived 2)-like 2 (Nrf2) gene, a key component of the oxidative stress signalling in animals [203]. This raises the possibility to use biotechnologically produced myconoside for medical applications. Furthermore, myconoside has also skin regenerative properties and can be used in cosmetics to improve wound healing and skin regeneration [17]. There is a lot of unexplored potential in studying secondary metabolites of the desiccation-tolerant plants which often live in specialized ecological niches.

## Conclusion

Since the first article documenting the systems biology approach for resurrection plants [182], the last years have resulted in a wealth of new information regarding the evolution of VDT, including several high-quality genomes, broad transcriptomic studies and some, still restricted, proteome and metabolite profiles. The results remain consistent with the hypothesis that VDT is derived from the DT mechanisms found in ancestral land plants, approximately analogous to the mechanisms identified in terrestrial green algae, bryophytes and other non-vascular plants discussed. Although the VDT trait is rare in angiosperms, genes of the desiccation response pathways appear to be common to all species given the lack of a significant genomic footprint between tolerant and sensitive species. Angiosperm VDT, then, can be understood as a lineage- or species-specific ability to reactivate these genes, resulting in a transcriptomic and metabolomic shift similar to that found in orthodox seeds and pollen. This is evidenced by the many examples of seed-restricted transcripts and metabolites that are abundantly expressed in vegetative tissues of DT plants. This implies that regulation of expression has lost the tight developmental control found in sensitive species, possibly through activation of dehydration responsive promoter elements or by re-programming the activity of transcription factors. In addition to de-regulating seed-specific expression patterns, VDT requires additional protective mechanisms of the photosynthesis apparatus—particularly in homoiochlorophyllous species—which may have been achieved by duplications and consequent abundant expression of genes with targeted protective functions, as it is hypothesized to have occurred with ELIPs. Nonetheless, although the core desiccation response appears to be conserved amongst green plants, there are differences in regulatory or metabolite profiles between species. These may be explained by differences in desiccation stress conditions (such as differences between rapid or slow water loss and the constitutive/inducible response, or between poikilochlorophyllous and homoiochlorophyllous species) or due to additional stressors related to the specific environment or physiology of the species. A systems-based approach must be applied to integrate these growing datasets and allow researchers to isolate the regulatory mechanisms that drive desiccation tolerance.

An ultimate goal coming from the study on resurrection plants is utilizing this knowledge for crop improvement. Deciphering the genetic and molecular networks responsible for desiccation tolerance may prove useful in understanding and controlling the regulatory mechanisms of drought stress tolerance in sensitive species. Activation of transcription factors regulating stress reprogramming and induction of stress-protective genes, such as ELIPs and LEAs, can contribute to tolerance to various abiotic stresses [204]. Manipulating the stress regulatory networks is a powerful approach for stress improvement. Furthermore, the expression of genes derived from resurrection species in desiccation sensitive species, including crops, has also been demonstrated to increase stress tolerance. For example, expression of osmotin from the resurrection plant *Tripogon loliiformis* in rice and tobacco conferred tolerance to cold, drought, and salt stress [205]. Expression of a glycosyltransferase gene from *Sporobolus stapfianus* in Arabidopsis resulted in enhanced growth and tolerance to both drought and cold stress [206]. More recently, expression of *XvSap1*, a gene related to cell wall stabilization in *Xerophyta viscosa*, enhanced drought tolerance in sweet potato [207]. Overall, these results indicate the vast and not yet fully explored potential of resurrection plants for crop improvement.

## Data Availability

Not applicable.
